# Insulin-Like Growth Factor 1 (IGF-1) Signaling in Glucose Metabolism in Colorectal Cancer

**DOI:** 10.3390/ijms22126434

**Published:** 2021-06-16

**Authors:** Aldona Kasprzak

**Affiliations:** Department of Histology and Embryology, University of Medical Sciences, Święcicki Street 6, 60-781 Poznań, Poland; akasprza@ump.edu.pl; Tel.: +48-61-8546441; Fax: +48-61-8546440

**Keywords:** insulin-like growth factor 1 (IGF-1), glucose metabolism, metabolic risk factors, colorectal cancer, Warburg effect, therapeutic strategies regulating insulin/IGF-1 signaling

## Abstract

Colorectal cancer (CRC) is one of the most common aggressive carcinoma types worldwide, characterized by unfavorable curative effect and poor prognosis. Epidemiological data re-vealed that CRC risk is increased in patients with metabolic syndrome (MetS) and its serum components (e.g., hyperglycemia). High glycemic index diets, which chronically raise post-prandial blood glucose, may at least in part increase colon cancer risk via the insulin/insulin-like growth factor 1 (IGF-1) signaling pathway. However, the underlying mechanisms linking IGF-1 and MetS are still poorly understood. Hyperactivated glucose uptake and aerobic glycolysis (the Warburg effect) are considered as a one of six hallmarks of cancer, including CRC. However, the role of insulin/IGF-1 signaling during the acquisition of the Warburg metabolic phenotypes by CRC cells is still poorly understood. It most likely results from the interaction of multiple processes, directly or indirectly regulated by IGF-1, such as activation of PI3K/Akt/mTORC, and Raf/MAPK signaling pathways, activation of glucose transporters (e.g., GLUT1), activation of key glycolytic enzymes (e.g., LDHA, LDH5, HK II, and PFKFB3), aberrant expression of the oncogenes (e.g., MYC, and KRAS) and/or overexpression of signaling proteins (e.g., HIF-1, TGF-β1, PI3K, ERK, Akt, and mTOR). This review describes the role of IGF-1 in glucose metabolism in physiology and colorectal carcinogenesis, including the role of the insulin/IGF system in the Warburg effect. Furthermore, current therapeutic strategies aimed at repairing impaired glucose metabolism in CRC are indicated.

## 1. Introduction

Insulin-like growth factor 1 (IGF-1), previously known as somatomedin C [1] is a hepatokine responsible for proper metabolic function of cells and the metabolism of the whole organism [2]. This liver-derived factor can be found in the circulation, serving a mainly endocrine function, with its production mainly controlled by pituitary growth hormone (GH, somatotropin) [3]. 

Metabolic IGF-1 functions mostly involve the maintenance of normal insulin sensitivity, glucose uptake increase, plasma triglyceride decrease, and cholesterol level regulation [2,4]. Furthermore, hepatic glucose metabolism may directly induce the transcription of the IGF-1 gene [3]. Both in physiological and pathological conditions, IGF-1 is subject to a complex genetic [5] and epigenetic regulation [6], which also influences the processes of carbohydrate and lipid metabolism. 

Circulating IGF-1 (cIGF-1) and its interactions with IGF Binding Protein 1 (IGFBP-1) are important determinants of glucose homeostasis, potentially indicating a protective role of IGF-1 against the development of glucose intolerance [7]. The impairment of IGF-1 synthesis results in a worsening state of insulin resistance (IR) [4]. Moreover, IGF-1 is responsible for the maintenance of hormonal balance between GH and insulin, with all three of these hormones crucial in maintenance of proper glucose metabolism [8,9].

Together with insulin, IGF-1 regulates the metabolism in response to the nutritional contents of the diet. Hypernutrition and hyperinsulinemia of obesity directly promote hepatic IGF-1 release and inhibit GH secretion [10,11]. Mechanisms of obesity formation and IR development [2], and the prevalence of metabolic syndrome (MetS), are not yet clear [12,13]. It is known that low levels of cIGF-1 predict the development of type 2 diabetes mellitus (DM II) and correlate with IR, increasing the risk of ischemic cardiovascular disease [7,14,15]. 

Mitogenic and anti-apoptotic activity of mature IGF-1, as well as different transcripts and precursor IGF-1 peptides, qualify IGF-1 to the group of growth factors implicated in the initiation and progression of various cancers, including colorectal cancer (CRC) [16,17,18,19,20].

Colorectal cancer is one of the most common human malignancies worldwide, with more than 1.9 million new CRC cases and 935,000 deaths in 2020. Overall, CRC ranks third in term of incidence, but second in terms of mortality [21]. Numerous epidemiological findings confirm the link between an increase in cIGF-1 and the risk of developing CRC [22,23,24]. Clinical data indicate an increased risk of CRC in patients with multiple environmental and life-style-related risk factors, including elevated visceral adipose tissue level [25], hyperglycemia [25,26] and/or obesity/overweight [26,27,28]. A metanalysis confirmed the relationship between such MetS components as higher values of body mass index (BMI)/waist, dysglycemia, higher blood pressure, and increased risk of cancer and mortality, regardless of the sex of the patient [29]. The association between MetS and the development of CRC involves the activation of signaling pathways associated with IR and the IGF system [30,31,32]. 

At the cellular level, changes in the metabolism of colonocytes may precede the acquisition of mutations leading to cancer transformation [33,34]. In turn, oncogenic mutations and loss of suppressor genes further reprogram CRC cells to upregulate glycolysis, glutaminolysis, one-carbon metabolism, and fatty acid (FA) synthesis. Primary cellular metabolism undergoes significant rearrangements during the initiation, growth and progression (including metastasis spread) of CRC (reviewed in: [35]). Compared to differentiated CRC cells, CD133^+^ colon cancer initiating cells favor glycolysis and lipogenesis [36]. Reviews highlight the close link between the changes in specific metabolic pathways in Lgr5+ intestinal stem cells (ISCs) and CRC initiation [35]. Hence, apart from cancer cells, metabolic changes also occur in cancer stem cells (CSCs) and tumor microenvironment (TME) cells and may have an impact on the host-microbiome crosstalk (reviewed in: [37]).

Hyperactivated glucose uptake and fermentation of glucose to lactate, even in the presence of functioning mitochondria, is known as the Warburg effect [38] and is considered as a one of six hallmarks of cancer [39,40,41], including CRC [42,43,44]. Although it is acknowledged that glycolysis occurs in CRC, the mechanism driving it remains largely unknown [33,40,42]. Glycolytic phenotype is an important part of the metabolic reprogramming of cancer cells and occurs at an early stage of oncogenesis, i.e., before the development of tissue hypoxia. The history and current knowledge of the mechanisms and consequences of the Warburg effect are presented in a recent excellent review [45]. On the other hand, the role of Insulin/IGF signaling in the acquisition of the Warburg metabolic phenotypes in colorectal cancer cells is still not fully described [33]. 

This review describes the role of IGF-1 in glucose metabolism in physiology and colon carcinogenesis, taking into account the role of Insulin/IGF system in Warburg effect. Current therapeutic strategies, aiming to repair impaired glucose metabolism in CRC, have also been indicated.

## 2. IGF System Components and Signaling

The IGF-1 signaling pathway plays an important role in maintaining the long-term health of many organisms. Among the components of this signaling pathway are two IGFs, IGF-1 and IGF-2 [46,47], six IGF Binding Proteins (IGFBP-1-6) [48,49], IGFBP proteases [50], as well as several other IGFBP-interacting molecules [51]. In physiology, they play an important role primarily in pre- and postnatal somatic growth and development [19,52,53]. IGFs present approximately 50% of sequence overlap with insulin, which classifies all of these proteins as members of the insulin superfamily, synthesized as prepro-proteins consisting of 4 domains (pre, B, C, A). Both IGFs belong to central hormones involved in metabolic signaling, affecting glucose uptake, lipogenesis, glycogen storage, and suppression of protein degradation [54,55]. Insulin-like effects of IGFs account for only 1-2% of insulin activity, with their main biological activity also involving stimulation of DNA, RNA and protein synthesis in tissue cultures [56]. In addition, unlike insulin of single-organ origin, the production of IGFs can take place in many locations across the human body. In addition, many tissues are sensitive to the local effects of this growth factor [46,57]. IGF-1 seems to be more structurally dynamic molecule [58] and has more potent mitogenic and anti-apoptotic activities than insulin [59]. Another distinguishing feature of IGFs is the presence of IGFs-specific binding proteins, the primary role of which is local modulation of IGF action. IGFBPs bind to their own receptors or move inside cells, where they are able to perform IGF-independent activities [48,49,60,61]. Binding to IGFBPs increases the half-life of IGFs in circulation and blocks their potential binding to the insulin receptor (INSR) [48].

Most of the cIGFs exists as ternary complex, where the half-life of IGF increases from 1-2 min for free IGFs, to more than 12 h. IGFs can also form binary complex with IGFBPs, increases their half-life to 20-30 min. IGFBPs, in addition to extending the half-life of IGFs, allow their storage in selected tissue compartments, inhibit their actions of by reducing the availability of receptors, as well as increase mitogenesis and cell migration as needed. The most common IGFBP circulating in the blood is IGFBP-3, which binds more than 95% of IGFs. This protein occurs in the form of dimer and forms a complex with acid-labile subunit (ALS) (reviewed in: [62]). 

IGFs have a direct effect on the cell by specifically binding to various membrane receptors: (i) type I (IGF-1R); (ii) type II (IGF-2R), (iii) INSR and (iv) hybrid receptor (IGF-1R/INSR) [60,61]. In most mitogenic and anti-apoptotic activities, both IGFs are mediated by IGF-1R, which can also independently affect cell transformation [61,63]. When ligands are bound to the corresponding receptors, they are activated by autophosphorylation via tyrosine kinase, stimulating various insulin/IGF-1 pathways. Among them, the two best characterized are the phosphatidylinositol 3 kinase (PI3K)/Akt/mammalian target of rapamycin complex (PI3K/Akt/mTOR) and the mitogen-activated kinase (MAPK) pathways, with the latter also involving Ras, Raf, MEK and ERK signaling molecules [16,64,65]. It is worth noting that mTOR signaling plays an important role in mitochondrial metabolism and is also a major downstream target of the 5′ adenosine monophosphate (AMP)-activated protein kinase (AMPK) signaling [66]. Through these signaling pathways, one of the effects of insulin/IGF-1 axis is the activation of cellular glucose uptake, which is then catabolized by glycolysis and/or oxidative metabolism, the latter providing the most efficient way of producing ATP. Increased glucose uptake occurs by increasing glucose transporter 1 (GLUT1) expression and glycolysis [39]. Analysis of the whole kinome (779 kinases) siRNA screen, showed an overexpression of 6-phosphofructo-2-kinase fructose-2,6-biphosphatase 3 (PFKFB3), a positive regulator of glycolysis in adipocytes and cancer cells, as a positive regulator of insulin/IGF-1 pathway [67]. 

### 2.1. IGF-1—Molecular Structure and Regulation

The human IGF-1 gene is composed of six exons, four of which are alternatively spliced [53,68,69]. All transcripts encode the same mature 7649 Da IGF-1 protein, made up of a single polypeptide chain containing 70 amino acids (AAs) and the so-called E-peptide at the 3’ terminus. Two and three E-peptides are present in rodents and humans respectively [70]. IGF-1 isoforms work through both common and unique pathways to promote biological effects. Local production of IGF-1 requires E-peptides to drive hypertrophy in growing muscles [68]. Both whole transcripts and the mature IGF-1 protein are detected in a wide variety of normal and tumor cells (reviewed in: [20]).

In the postnatal period, hepatocytes are the main source of IGF-1 in response to GH. Hepatic IGF-1 has endocrine effects, while IGF-1 synthesized by cells of other organs acts para- and/or autocrinally [5,71,72,73]. Organs such as kidneys, lungs, heart, gonads [74], brain, thyroid, large intestine [17,75,76], as well as cells of lymphatic organs (e.g., monocytes/macrophages, NK cells, T and B cells) [77,78,79] are able to produce IGF-1 (mRNA and protein) and demonstrate the presence of IGF-1R.

IGF-1 has a diverse effect on cells and tissues [1,57]. It performs important roles in the development of organs and tissues, their postnatal growth, regulates homeostasis of mature muscle tissue, and determines the survival of the body [47,80]. The main target of IGF-1 are skeletal muscles, where it elicits its insulin-sensitizing effects [8]. The processes intensifying local muscle tissue growth through IGFs are related to glucose homeostasis and insulin signaling. In addition, muscle IGF-1 (mIGF-1) has been shown to independently modulate anabolism and metabolism in an age-dependent manner. According to the authors, maintenance of mIGF-1 is critical for both muscle growth and metabolic homeostasis [80]. IGF-1 can directly stimulate glucose transport to muscles through IGF-1 receptors or hybrid IGF-1R/INSR receptors [81]. IGF-1 also has a direct effect on bone growth, with indirect bone action being the result of its direct effects on muscles. IGF-1 is crucial for bone development and the maintenance of bone mass. The complex interactions between muscle and bone through IGF-1 have already been reviewed [82]. 

On the other hand, several factors have a regulating effect on the synthesis and secretion of IGF-1. In vitro studies of rat fetal hepatocytes supported the hypothesis that the IGF protein family is positively regulated by glucose at gene level. Glucose-induced IGF-2 mRNA (the main IGF in fetal developmental stages) was mediated by stimulation of gene transcription and increased transcript activity [83]. During embryonic development, it was proven that insulin and nutrients have a bigger influence on IGF system regulation than GH. Insulin mediated increase in both IGFs occurs due to the transcript stability increasing effect of this hormone rather than an increase in gene transcription [84]. In turn, transcription of the IGF-1 gene directly may be influenced by glucose metabolism in the liver [3] and its disorders [85]. Similarly, hepatic uptake of certain AAs, induced by GH or insulin, facilitates the expression of IGF-1 by stabilizing IGF-1 mRNA [3]. A new mechanism for arginine-mediated IGF-1 secretion regulation in hepatocytes has recently been described. First, GH stimulates IGF-1 translation and increases levels of IGF-1 protein, leading to its secretion. Then, arginine induces the release of IGF-1 from the endoplasmic reticulum (ER), which implies it in the process of IGF-1 retention in the ER [86].

In addition to insulin or GH, the production and secretion of IGF-1 are also influenced by other hormones, e.g., sex hormones [87], as well as factors such as age [19,88], sex, diet and nutrition [89], circadian rythm [90], and microbiota-derived metabolites [91]. Genetic defects with potential to affect both GH secretion and IGF-1 bioavailability and action are also a subject of a recent review [92]. The reduction in IGF-1 production occurs primarily in chronic liver diseases, e.g., viral-associated chronic hepatitis, and hepatocellular carcinoma (HCC) [93,94,95]. In the HCC mouse model, the pathogenesis of HCC was shown to be accompanied by e.g., a reduction in IGF-1 and reprogrammed metabolic profiles shifted towards increased glycolysis and lipogenesis [94]. The concentration of IGF-1 also depends on the quantity and quality of nutrition. It increases under the influence of a high-protein and high-fat diet and decreases in a diet rich in carbohydrates. Malnutrition results in reduced hepatic IGF-1 production and decreased cIGF-1 levels, resulting in an increase in GH in a feedback loop [96].

### 2.2. IGF-1 and Glucose Metabolism

While IGF-1 is mainly classified as a mitogenic and insulin as a metabolic hormone, they both participate in regulation of glucose homeostasis [8,64,65]. IGF-1 also plays a role in lipolysis, proteolysis, and protein oxidation [97], of which the effect on lipolysis is the least conclusive. IGF-1 has been shown to significantly increase the effect of GH on lipolysis and ketogenesis (increase in non-esterified FA (NEFA), glycerol, and 3-OH-butyrate levels), as well as disposal of ketone bodies by skeletal muscle [97]. Other studies indicate an IGF-1-mediated reduction in NEFA concentration [98], as well as lack of this protein’s effect on glycerol production rate, an index of lipolysis in DM I [64]. It has also been shown that GH itself is likely to have a direct effect on lipid metabolism in IGF-1-independent mechanisms [99]. Compared to insulin, IGF-1 inhibits lipolysis less effectively [100]. 

IGF-1 also has opposite effects on carbohydrate, lipid and protein metabolism compared to GH [52,101,102]. Excess GH causes IR and hyperglycemia, whereas IGF-1 reduces blood glucose levels [102]. 

Together with insulin, IGF-1 interacts with related receptors to lower plasma glucose, evidenced by comparative studies of these two hormones. Thus, while the use of higher than physiological concentrations of recombinant human (rh) IGF-1 and insulin in healthy volunteers, showed the lowest and comparable decrease in glucose levels after 30 min from bolus intravenous peptide administration, IGF-1 was only 6% as potent as insulin in the inducing of hypoglycemia [100]. Other studies of acute rhIGF-1 infusion compared to insulin showed a preferential increase in peripheral glucose utilization, diminished suppression of hepatic glucose production, and augmented decrease of whole-body protein breakdown (leucine flux), but comparable antilipolytic effects. The insulin-like effect of IGF-1 is, according to the authors, mediated in parts via IGF-1Rs and INSR [103]. In another study physiological doses of rhIGF-1 also increased glucose utilization (glucose disposal) and directly suppresses insulin secretion. This reduction in insulin levels and inhibition of GH via rhIGF-1 resulted in increased insulin sensitivity [104]. It has been confirmed that IGF-1 is necessary for insulin sensitivity in physiology [54], in DM I [64,105], as well as in DM II [106]. Direct IGF-1 effects on glucose metabolism in DM I have also been proven, through a reduction of hepatic glucose production and increase in peripheral glucose uptake, which has been maintained during a hyperinsulinemic euglycemic clamp [64]. In addition, it has been shown that the complex of rhIGF-1 and IGFBP-3 increased glucose metabolism by controlling both endogenous glucose output and peripheral glucose uptake. These studies confirm the role of IGF-1 in regulating insulin sensitivity both directly and indirectly through GH suppression [105]. 

The direct effects of IGF-1 on glucose metabolism are mediated by the effect of IGF-1 on pancreatic insulin secretion or glucose uptake by muscle. On a mouse model with liver-specific *igf-*1 gene deletion (LID model), very low concentrations of cIGF-1, high concentrations of GH and hyperinsulinemia were observed, associated with muscle insulin insensitivity. Therefore, this model of research has shown that low levels of cIGF-1, causing excessive GH secretion, lead to an increase in insulin activity in peripheral tissues (muscles) in vivo [8]. Hence, a metabolic role of cIGF-1 is also suggested [8,9,107]. 

Insulin and IGF-1 are nutritionally regulated hormones that reset circadian clocks by inducing Period proteins, while abnormal insulin signaling in vivo and in in vitro results in disruption of clock gene expression and the circadian organization of mouse behavior [108]. Chaudhari et al. showed that IGF-1 level in blood and IGF-1 signaling in vivo demonstrates circadian rhythms [90]. 

Although the role of insulin and IGF-1 in regulating metabolism in response to nutrients is well known, the role of IGF-1 in obesity mechanisms and IR is less described [54,109]. Both dietary protein content and energy intake regulated cIGF-1 concentrations in adult volunteers, with energy intake showing a potentially greater importance [109]. 

An inverse correlation between IGF-1 and MetS and its consequences is suggested. The mouse model showed that only partial IGF-1 deficiency is responsible for reducing the liver expression of genes involved in glucose and lipid metabolism, leading to hyperglycemia and dyslipidemia. IGF-1 induces the opposite effects than insulin since IGF-1 deficit reduces the expression of genes involved in glucose metabolism, e.g., glucose-6-phosphatase, catalytic (*g*6*pc*) and phosphoenolpyruvate carboxykinase 1 (*pck*1). Thus, it appears that the actions of IGF-1 are antagonistic to insulin rather than “insulin-like” [13].

Significantly lower IGF-1 levels have been shown in MetS compared to patients without this syndrome, making this factor a potential marker of IR [12]. The effect of IGF-1 on metabolism (especially of carbohydrates and lipids) and the implications of IGF-1 deficiency in the establishment of MetS were already also reviewed [110]. 

## 3. IGF-1 and Colorectal Cancer Pathogenesis

The potential link between serum concentrations, tissue expression of IGF-1 (including different mRNA isoforms) in colon carcinogenesis was presented in our earlier reviews [20,111]. Most epidemiological studies highlight the role of cIGF-1 as a risk factor for the development and progression of CRC [22,24,112,113,114]. As a rule, higher concentrations of cIGF-1 and reduced cIGFBP-3 were associated with an increased risk of CRC in both men and women [112,115,116], which is also confirmed by complementary serologic and Mendelian randomization analyses [24]. However, a meta-analysis along with prospective studies by Rinaldi et al. indicated a relatively modest association of CRC risk with serum IGF-1 levels [117]. 

Chronically elevated fasting and postprandial insulin and IGFs levels increase the risk of CRC through various mechanisms. One of these may be lowering the concentration of cIGFBP-1 and cIGFBP-2, resulting in an increase in plasma levels and bioavailability of free IGF-1 [118,119]. 

Tissue expression studies of IGF-1 (mRNA, protein) in CRC show different rates of positive tumors, from a few % to as much as 80% [120,121,122,123] or present negative results [124,125]. In quantitative terms, local production of IGF-1 (mRNA, protein) in CRC vs. non-cancerous tissues is also very diverse. Some authors report that IGF-1 mRNA expression is higher in CRC vs. adjacent nontumor tissues [123], while other studies have shown lower expression of this transcript in CRC vs. adjacent normal colon mucosa [121,126]. 

Comparison of serum IGF-1/IGFBP-3 concentrations (ELISA methods) in selected epidemiological studies in different stages of CRC and tissue expression of IGF-1, IGF-1R, and IGFBP-3 based on immunohistochemical or PCR techniques shows wide variation in serum concentrations and local expression of IGF axis components in vivo depending on studies, number of patients studied, and relatively poor correlation with clinical data [Table 1]. 

The mouse CRC model has also shown positive effects of cIGF-1 on the development and metastasis of CRC to the liver. Administration of human rIGF-1 to both LID animals and the control group increased cIGF-1 and cIGFBP-3 levels in both animal groups. In addition, correlations between cIGF-1 and vascular endothelial growth factor (VEGF) expression and blood vessel density in the cecum tumors were demonstrated [18]. 

Numerous in vitro as well as in vivo studies confirm that both normal colonocytes and CRC cells have IGF-1 receptors and INSRs [120,127,128,129,130,131]. They can be activated both by IGFs and by insulin. However, it is now known that most insulin effects occur through direct interactions with INSR [132]. Activation of all these receptors results in an increase in cell cycle progression, proliferation and inhibition of cell apoptosis [127,128,129,130]. Pro-proliferation effects of both IGFs were confirmed in five out of eight human CRC cell lines [133].

### 3.1. IGF-1 and Glucose Metabolism Disorders as Risk Factors for Colorectal Cancer

#### 3.1.1. Metabolic Syndrome (MetS)

MetS is one of the major metabolic diseases, alongside DM II and non-alcoholic fatty liver disease (NAFLD). It is diagnosed in patients with at least three of the following factors: abdominal obesity, hypertriglyceridemia, increased blood pressure, low high-density lipoprotein cholesterol (HDL) and/or glucose intolerance [134]. 

A link between MetS and CRC has already been demonstrated [29,32], with a meta-analysis by Esposito et. al. confirming that MetS is associated with an increase in CRC incidence and mortality in both men (RR: 1.33, 95% CI, 1.18–1.50, and 1.36, 1.25–1.48, respectively), and women (RR: 1.41, 1.18–1.70, and 1.16, 1.03–1.30, respectively). However, individual MetS factors, particularly higher BMI/waist and/or dysglycemia in these patients may have explained the increased risk of CRC [29]. In turn, a meta-analysis of Crawley et al. showed a positive correlation between serum glucose and cancer risk. However, based on studies involving heterogeneous human cancers (including CRC), the mechanisms of this correlation, could only be partially explained by changes in the insulin/IGF-1 axis [135]. 

For CRC, a modest increase of in situ risk was demonstrated in patients with fasting plasma glucose levels > or = 116 mg/dL, supporting the hypothesis that hyperinsulinemia may play a role in colorectal carcinogenesis [136]. Schoen et al. demonstrated that patients in the highest quartile of fasting glucose had a ~twofold increased risk of CRC [25]. In addition, it has been shown that higher serum glucose levels were more strongly associated with increased risk of cancer relative to controls (OD, 3.0; 95% CI, 0.9–9.8) than with increased risk of advanced adenoma (OD 2.1; 95% CI, 0.9–5.4) [26]. On the other hand, Limburg et al. demonstrated that the highest quartiles for each biomarker, insulin, and HOMA-IR were associated significantly with CRC incidence, whereas glucose was marginally linked to CRC risk (HR, 1.70; 95% CI; 0.92–3.13) in age-adjusted models. However, the authors are quite cautious in considering hyperinsulinemia, hyperglycemia, and/or IR as risk factors for CRC in the male smokers examined in this prospective study [137]. Gunter et al. described two independent pathways related to colon carcinogenesis in postmenopausal women. One of them included higher levels of endogenous estradiol, while the other was associated with obesity, hyperinsulinemia and free IGF-1 levels [28]. On the other hand, later studies of this team, conducted in postmenopausal women, suggested that elevated serum glucose, rather than insulin level and homeostasis model assessment, may be a risk factor for CRC [138]. Similarly, there was no link between plasma insulin levels, HOMA2-IR and CRC in studies by Vulcan et al. [139].

Systematic review and meta-analysis of Xu et al. showed that higher levels of glucose and fasting insulin, HOMA-IR, glycated hemoglobin (HbA1c) and peptide C were significantly associated with an increased risk of developing CRC. Furthermore, HOMA-IR appears to be a better indicator of CRC risk than glucose or fasting insulin [140].

#### 3.1.2. Obesity

Early work on IGF-1 concentrations in obesity showed that IGF-1 levels are inversely proportional to BMI [96]. Although total IGF-1 levels in the blood are lowered in obese people, the concentration of its free fraction is elevated, possibly due to hyperinsulinemia [141].

Obesity is associated with IR, compensated by an increase in insulin production by β cell of pancreatic islets and the resulting hyperinsulinemia. The insulin/IGF-1 system is one of the potential “engineers” of many obesity related cancers [142]. The meta-analysis showed a link between increased pre-diagnostic blood levels of insulin and glucose and the development of pancreatic and colorectal cancers [143]. Although insulin does not have mitogenic properties, its excessive increase can be a signal for tumor growth and its aggressiveness, including CRC [28]. In hyperinsulinemia, an up-regulation of GH receptor and hepatic IGF-1 synthesis can be observed [144]. In addition, chronic hyperinsulinemia lowers the hepatic expression of IGFBPs (BP-1 and BP-2), resulting in increased plasma levels and bioavailability of free IGF-1 [11,118]. Insulin appears also to increase the ratio of circulating IGF-1/IGFBP-3 by increasing hepatic sensitivity to GH [119].

Numerous animal models have shown that caloric restrictions are associated with a decrease of IGF-1 serum levels, resulting in the anti-tumor and anti-aging activity of IGF-1 (reviewed in: [145]). In turn, the mouse model of high fat diet-induced obesity showed a significant increase in hypothalamic IGF-1 expression as compared to mice fed with a standard chow diet. It has also been shown that in mice fasted for 4 or 16 h, IGF-1 expression in brain structures after “acute” central IGF-1 treatment or prolonged IGF-1 overexpression results in a significant increase in insulin serum and reduced blood glucose levels. In both conditions, improved glucose tolerance and enhanced insulin sensitivity were observed. Overexpression of IGF-1 in the brain led to increased phosphorylation of INSR (INSRb subunit) and Akt, a key signaling pathway stimulated by IGFs. Additionally, decreased pro-opiomelanocortin (*Pomc*) levels in the hypothalamus, and increased uncoupling protein 1 (UCP1) expression in brown fat tissue were observed. The authors conclude that central IGF-1 promotes feeding, improves glucose tolerance and insulin sensitivity, and can stimulate energy expenditure via thermogenesis [146].

The role of waist-to-hip ratio (WHR), combined with changes in serous concentrations of IGF-1 and IGFBP-3, was also indicated during the initiation and progression of CRC [114]. IGF-1 is further known to belong to adipokines, together with leptin, insulin, interleukin 6 (IL-6), also produced by fat cells. This may result in an etiological relationship with the occurrence of CRC, sometimes related to as an obesity-associated cancer [147]. In addition, as the study of Succurro et al. conducted on more than 100 nondiabetic subjects with a wide range of BMI values showed, progressive reductions in IGF-1 concentrations may be involved in obesity-related changes in both insulin sensitivity and secretion [15]. 

#### 3.1.3. Diabetes Mellitus (DM)

Loss of functional pancreatic islet β-cell mass leads to a deficiency in insulin secretion, leads to a deficiency in insulin secretion, the development of DM I and DM II, and metabolic disorders characterized by high blood glucose levels [148]. In patients with incorrectly controlled DM I and patients with DM II treated with insulin, there is a decreased level of IGF-1 in the blood due to reduced hepatic production of this growth factor [96,149]. 

The link between DM II and cancer development has long been discussed, and the mechanisms of elevated glucose concentrations as a carcinogen are still being studied [144]. Increased blood glucose (both with and without a history of diabetes) [150] is significantly associated with an increase in CRC risk in both sexes [150,151], or only in men [139]. 

Biological mechanisms linking diabetes to cancer include both hyperglycemia and hyperinsulinemia, increased bioactivity of IGF-1, oxidative stress, dysregulations of sex hormones, and chronic inflammation [152]. One of the suggested signaling pathways in these mechanisms is the Insulin/IGF system. IR, which causes insulin dysfunction in DM II or MetS, leads to prolonged hyperinsulinemia. This reduces the production of IGFBPs, which consequently increases IGF-1 levels resulting in increased proliferation and inhibition of cell apoptosis [11,153]. 

Studies show that ~12–20% of diabetic patients are at risk of CRC and the incidence rate is more than twice as high as in other populations [22,154]. Hyperglycemia and DM (mainly type 2) are associated not only with a higher incidence, but also with the progression (including mortality) of CRC [155,156,157,158], as also confirmed by older and recent meta-analyses [159,160,161]. Some authors show that the risk of CRC among obese people with diabetes increases with longer duration of obesity of 4–8 years (HR 1.19; 1.06–1.34) and >8 years (HR: 1.28; 1.11–1.49) [157]. Retrospective studies of Han et al. confirmed an increased incidence of CRC in diabetics compared to non-diabetic controls. The CRC group of diabetic patients had higher serum IGF-1 and IGF-1 mRNA levels and lowered IGFBP-6 levels. Similarly, higher tissue expression of IGF-1 and IGF-1R, as well as lower levels of IGFBP-6 in CRC vs. adjacent healthy tissues were observed [162]. Concomitant diagnosis of CRC and DM was associated with an increased risk of overall (HR, 1.21; 95% CI, 1.17–1.25) and cancer-specific mortality (CSM) (HR, 1.11; 95% CI, 1.05–1.17), as well as an increased risk of cancer recurrence (HR, 1.09; 95% CI, 1.02–1.16) [161].

The role of insulin/IGF-1 system in the development of both DM and CRC is indisputable [158,163]. Insulin, as a strong growth factor, appears to promote cell proliferation and carcinogenesis directly or through IGF-1 [132]. As mentioned, hyperinsulinemia leads to an increase in IGF-1 bioactivity through inhibition of IGFBP-1 and IGFBP-2 [11,119]. In addition, high glucose levels can have a direct and indirect effect on cancer cells, promoting their proliferation. Growth of the tumor may therefore occur in response to insulin, glucose and both IGFs (reviewed in: [155]). These data are also confirmed in studies in CRC tissues with DM II, as well as in selected animal models. Ding et al. showed that a history of diabetes in CRC patients was associated with tissue expression of the main components of the IGF axis, and that higher tumor (T) stage and lymph node metastases were respectively independent factors of IGF-1 and IGF-1R expression in these patients. In addition, higher expression of IGF-1 and both receptors (IGF-1R and INSR) in CRC patients was associated with DM [156]. In turn, Liu et al. demonstrated increased mRNA production of IGF-1 and IGF-1R in cancer tissue vs. non-cancerous tissue in CRC patients with and without DM II. Higher IGF-1 transcription was observed in the CRC group with DM II. Furthermore, IGF-1 mRNA was also a risk factor for CRC prognosis [163]. 

In the mouse model with CRC and DM II transplants, increased tumor mass was observed in the diabetic group compared with CRC alone. In addition, the diabetes group showed higher concentrations of serum IGF-1 compared to control and CRC group. An increase in tissue expression of VEGF was also observed in the CRC-DM II group. Hence, DM II would be the mechanism for CRC promotion, while IGF-1, by induction of VEGF gene transcription, would be responsible for angiogenesis resulting in CRC metastasis [154]. 

The role of cIGF-1/cIGFBP-3 in colonic stem cells (CoSCs) function and their dysfunction in diabetic enteropathy in DM I has also been confirmed. Gene expression analysis suggests that hyperglycemia and circulating growth factors jointly alter the self-renewing properties of CoSCs in long-term DM I. The effect of high glucose on hepatic release of IGFBP-3 in the supernatant of human immortalized hepatocytes has also been confirmed. Restoration of normoglycemia in patients with long-standing DM I normalized cIGF-I/cIGFBP-3 levels and restored CoSC homeostasis [164]. 

Crossing of C57BL/KsJ-db/db (db/db) mice, obesity model and DM II, and C57BL/6J-ApcMin/+ (Min/+) mice, a family model of adenomatous polyposis, resulted in creation of three mouse strains, the db/db-Min/+, db/m-Min/+, and m/m-Min/+ mice. Significant increases in insulin, cholesterol and triglyceride concentrations and an increase in mRNA levels of both IGFs and IGF-1R were shown in db/db-Min/+ mice compared to db/m-Min/+ and m/m-Min/+mice, which promoted the formation of numerous intestinal adenomas. These studies suggest that hyperinsulinemia in db/db-Min/+ mice activates signaling cascades involving IGF-1R, resulting in a proliferative response [165]. 

The role of insulin/IGF-1 signaling pathways in maintaining a differentiated phenotype of pancreatic islet β-cells and the development of obesity-associated DM II is discussed. According to the authors, hyperinsulinemia-mediated islet and β-cell insulin/IGF-1 resistance may be involved in the decomposition of β-cells [166]. 

Low concentrations of IGF-1 were proven to be strictly linked to growing risk of glucose intolerance and the development of DM II [7]. The rat model of DM showed a significantly reduced level of cIGF-1, and a decrease in the expression of two IGF-1 transcripts (IGF-1a and IGF-1b) in the liver, kidneys, and lungs in these animals. The constant value of IGF-1a/IGF-1b ratio suggests that post-transcription splicing is not affected by either diabetic state, chronic GH hyperstimulation, or insulin therapy. However, reduced concentrations of cIGF-1 and a decrease in the availability of this peptide in tissues may be responsible for the growth retardation seen in uncontrolled DM [167]. 

Hyperinsulinemia, which can contribute to common defects in insulin/IGF-1 pathways on the pancreatic periphery and in the pancreatic islet β-cell, is considered a key causative factor for the development of DM II [166]. According to the authors, long-term exposure to high insulin levels may induce not only IR of β cells, but also resistance to IGF-1, which may contribute to β cell failure in DM II. It is suggested that insulin initially works to increase its own secretion in response to stimulation of excess glucose and glucagon-like peptide (GLP) [166,168]. However, when local insulin levels become high, its secretion is inhibited [166]. Increased insulin secretion in vivo stimulates IGF-1 synthesis in the liver, with negative feedback inhibiting insulin secretion from β-cells via IGF-1R signaling, as well as possible hybrid INSR/IGF-1R receptor binding [168,169]. 

#### 3.1.4. Acromegaly

In acromegaly, which is most often caused by pituitary adenoma, hypersecretion of GH occurs, resulting in a further increase of cIGF-1 [170,171]. Metabolic characteristics of active acromegaly include impaired glucose tolerance, DM and IR even though both GH and IGF-1 are elevated [170,172,173,174]. Abnormal glucose metabolism has been shown to occur more frequently in patients with pure somatotroph adenomas than those with mixed adenomas. The relationship between impaired glucose metabolism and pituitary pathology persists even after normalization of IGF-1 levels [172]. IGF-1 rather than GH was a significant risk factor for glucose intolerance after adjusting for clinical data. In addition, in this disease it was IGF-1, not GH, that correlated more closely with IR [173]. In another study, IGF-1% upper limit of normal (ULN)/GH ratio was used to assess the effect of glucose metabolism disorders on IGF-1 levels. A reduction in this ratio was demonstrated in patients with IR (HOMA-IR > 2.5) or prediabetes [85]. Similarly, Maione et al. demonstrated a positive correlation between baseline ULN IGF-1 levels and DM II, even after adjustment for age, BMI, and both factors [174]. IR, hyperinsulinemia and increased gluconeogenesis, which combine to produce a metabolic milieu all play a role in the pathogenesis of DM in acromegaly, and are also associated with the pathology of the large intestine in this disease [175].

As research shows, one of acromegalic comorbidities are different malignancies, including CRC [174,176,177]. Fasting insulin and HOMA-IR levels correlate positively with hyperplastic polyps and adenomas in patients with acromegaly with divisive changes in the large intestine [178]. A link was also investigated between acromegaly, increased levels of GH/IGF-1 and higher risk of colorectal neoplasia [179,180,181]. Early development of new adenoma (but not hyperplastic polyps) was shown to be associated with both elevated serum IGF-1 levels and previous adenoma during primary colonoscopy [179]. Significant risk factors for colon neoplasia in acromegalic patients are still believed to be an adenoma on initial screening, as well as elevated serum concentrations of GH or IGF-1, i.e., uncontrolled acromegaly [179,181]. 

The incidence of colon polyps in patients with acromegaly ranges from 6–30% for both adenomatous and non-adenomatous lesions, with the incidence of CRC varying from 4–10% [181]. In a study by French authors, cancer occurred in 10% of acromegalic patients with incidence ratio of 1.34 in men, and 1.24 in woman. Regarding the GH/IGF-1 levels, it was demonstrated that ULN IGF-1 (but not GH) levels correlated with the presence of polyps, even after adjustment for age, BMI and smoking, both separately and together. Neither GH nor ULN IGF-1 levels have been associated with cancer or with tumor site in these patients [174]. In contrast, in Italian authors’ studies, the presence of polyps was significantly associated with both GH and IGF-1 levels, fasting glucose and insulin levels. Polyps and adenomatous polyps were more common in patients with acromegaly than in the control population [182]. Other studies indicate that the presence of hyperplastic/adenomatous polyps depends on both the occurrence of previous polyps in colonoscopy and elevated IGF-1 levels [183]. The link between acromegaly and the development of benign and malignant CRCs (mainly adenocarcinoma) has also been confirmed in the Japanese cohort study [184]. Such a link is also supported by the only available meta-analysis [185], as well as most recent multicenter case-control retrospective study [177]. The first work showed that patients with acromegaly have an increased risk of developing both colorectal adenomatous and hyperplastic polyps as well as CRC. For CRC, OR was 4.35 (95% CI, 1.53–12.35). Some controversies regarding the increased risk of CRC in acromegaly are explained by the large heterogeneity of study design and the lack of an ideal control group among the studies evaluated in this meta-analysis [185]. 

A recent study on 70 acromegalic patients confirmed an increased risk of preneoplastic colonic alterations and CRCs in patients with chronic and sustained GH excess vs. control group. IR, on the other hand, was the only statistically significant factor in acromegalic patients with and without colonic polyps [177]. The increased risk of colorectal neoplasia in acromegalic patients is due to e.g., excessive production of GH and IGF-1, resulting in increased proliferation of colon cells and decreased apoptosis ratio. Elevated levels of IGF-1 were associated with increase proliferation in the superficial crypt cells [186]. In addition, normal and CRC cells may overexpress IGF-1R or GH-R (reviewed in: [170]). 

Due to the still unclear mechanisms of colorectal neoplasia in acromegaly, these patients are recommended routine screening starting at acromegaly diagnosis, followed by appropriate surveillance depending on findings from initial colonoscopy and disease activity [176]. 

The main relationships between insulin and IGF-1 and glucose metabolism in different research models are shown in Table 2. 

### 3.2. IGF-1 and Glucose Metabolism in Normal Colonocytes and CRC Cells

Glucose is the main anabolic and catabolic substrate and regulator of numerous metabolic pathways in the normal cell, with its metabolism widely accepted as a major contributor to cancer development. Glucose is responsible for many functions, e.g., gene transcription, enzyme activity, hormone secretion or glucoregulating neuron activity. As a signaling molecule, it requires numerous transporters (GLUTs) to catalyze the facilitated two-way transfer of its substrates through cell membranes. Isoforms of GLUTs 1–4 with different regulatory and/or kinetic properties are well described. Various glucose functions are usually secondary to glucose uptake, which in most tissues (with the exception of hepatocytes and pancreatic β cells) is controlled by the level of GLUT expression on the cell surface (reviewed in: [187]). 

In normal cells of the body, glucose is processed through oxidative phosphorylation (OXPHOS), while glycolysis only becomes the main way to metabolize it under hypoxia conditions [37,42]. 

In cancer cells, abnormal glucose metabolism occurs, known by the name of the discoverer, Otto Warburg, the winner of the Nobel prize in medicine in 1931, as Warburg effect [38,39]. According to this phenomenon, in cancer cells, the dominant pathway of glucose metabolism is glycolysis, even in the presence of oxygen, followed by fermentation of glucose into lactate [38]. Glucose is therefore not used to synthesize ATP in mitochondrial OXPHOS. ≥10 times increased glucose uptake, GLUTs deregulation and increased glycolysis rate are observed, with aerobic glycolysis used to produce ATP in a relatively inefficient way [39]. As a result, cancer cells need to burn huge amounts of glucose in order to grow and multiply. The Warburg effect is a mechanism that primarily supports the survival of cancer cells in conditions of different availability of oxygen and nutrients and is an early event in oncogenesis. It promotes proliferation, tumor growth, as well as the acquisition of chemoresistance by tumor cells [39,41,44,45]. GLUT1 is particularly important in regulating cancer cell proliferation and invasive metastatic potential through glycolysis mediation, while in the case of rectal cancer it is also a bad prognostic factor for disease-free survival (DFS) [188].

Originally, the Warburg effect was associated with mitochondrial damage, with a recent resurgence in the popularity of this theory, as it is believed that this effect may also be caused by mitochondrial dysfunction in tumors [45]. In addition, it is known that increased lactate synthesis occurs not only from glucose in aerobic glycolysis, but also from glutamine by glutaminolysis, with both processes occurring in normal mitochondria [37,40,45]. It was discussed whether the Warburg effect plays a causal role in the formation of tumors or whether it is an epiphenomenon in tumorigenesis [189]. Currently, it is believed that in most cancers Warburg effect is the result of normoxic or hypoxic interaction of HIF-1 overexpression, activation of oncogenes (c-Myc, Ras), loss of tumor suppressor function (mutant p53, mutant phosphatase and tensin homolog on chromosome ten (PTEN), microRNAs, and sirtuin), activation (e.g., PI3K/Akt/mTORC1, Ras-Raf-MEK-ERK-cMyc, and JAK/STA3) or deactivation (e.g., AMPK) of signaling pathways, TME components and interaction of HIF-1 with epigenetic mechanisms (reviewed in: [45]).

As recent research suggests, most CRC cells exhibit Warburg metabolic phenotype [37,42]. It should be recalled that normal colonocytes are unique in their use of butyrate (BT) rather than glucose as the primary source of energy [190,191]. In contrast, colorectal epithelial cancer cells show a decrease in BT uptake through a reduction in the expression of monocarboxylate transporter 1 (MCT1) and sodium-coupled monocarboxylate transporter 1 (SMCT1). However, the rate of glucose uptake increases, and glycolysis becomes their main source of energy, which is considered a consequence of the Warburg effect [189,191,192,193]. The opposite effect of BT on the growth of normal (potentiation) and cancerous colonocytes (inhibition) is known as the BT paradox [192,193].

Change of primary energy source from BT to glucose, resulting in glycolytic phenotype [39], is a much faster way of producing ATP for CRC cells, essential for increased cellular proliferation and tumor growth. Such a phenotype facilitates the cellular use of glycolytic intermediates for the synthesis of macromolecules required to support proliferation. This is confirmed by studies in CRC tissues that have shown increased tissue expression of lactate [194,195], lactate dehydrogenase A (LDHA) [196], LDH5 [197,198], and hexokinase II (HK II) [199] as compared with normal tissues. Knockdown of LDHA resulted in reduced lactate and ATP production and glucose uptake [196]. LDH5, as one of the five isoenzymes of LDH and, seemingly, the most important in promoting anaerobic glycolysis, was also correlated with the increased regulation and accumulation of HIF-1α and HIF-2α, and they were all associated with aggressive phenotype in CRC [197]. Satoh et al. indicated glucose as the second most reduced metabolite in tumor tissue, with lactate levels exhibiting significant elevation, suggesting glycolysis activation. In addition, metabolic changes were shown to occur both at the adenoma stage and early in the adenoma-carcinoma sequence of CRC progression. They remained present through all cancer stages and were not associated with mutations of typical genes involved in colon carcinogenesis, e.g., adenomatous polyposis coli (*APC*), tumor protein 53 (*TP53*), and *KRAS*. In contrast, the aberrant expression of the MYC gene activated glycolysis by increasing the expression of glycosylphosphatidylinositol (GPI), ATP-dependent 6-phosphofructokinase, muscle type (PFKM), enolase 1 (ENO1), LDHB, and a decrease in expression of phosphoenolpyruvate carboxykinase (PEPCK), an enzyme that reduces the rate of gluconeogenesis, suggesting that MYC expression induces the Warburg effect [195]. Mizuno et al. demonstrated topographically differentiated overexpression of metabolic enzymes, where up-regulated glutaminase (GA) was mainly located at the invasive margin, elevated LDHA mainly at the center of the tumor, and HK II evenly in both locations [199]. 

#### The Role of Insulin/IGF-1 System in Glycolytic Phenotype of CRC Cells

CRC metabolic tumors, classified as consensus molecular subtype 3 (CMS3) (13% of all CRCs), are characterized by chromosome instability (CIN), frequent *KRAS* mutations (68%), and dysregulation of metabolic pathways (including glucose and fructose) [44,200]. The aerobic glycolysis process increases the share of numerous genes associated with glucose metabolism, e.g., hypoxia-inducible transcription factor (HIF), glucose-regulated protein 78 (GRP78), yes-associated protein 1 (YAP), cellular prion protein (PrPc), and estrogen-related receptor α (ERRα) and the regulation of many types of miRNA (reviewed in: [43]). 

The effect of insulin and/or IGF-1 on the formation of glycolytic phenotype in CRC has also been studied. In various cultured human colon adenocarcinoma cells, an increase in glucose consumption was observed, although mechanisms varied. Insulin directly influenced the use of substrates by the glycolytic pathway, but without affecting the activation of the glucose transport pathway in HT29 cells [201]. Neither insulin nor IGF-1 affected glucose transport or lactate production by another line of CRC cells (Caco-2). Receptors for insulin and IGF-1 in Caco-2 cells have been found not to regulate glucose transport. Glucose absorption by monolayer occurred via Na+/glucose cotransporter [202]. Studies on HT29-D4 cells, on the other hand, showed that IGF-1 significantly increased the initial rate of glucose uptake. In addition, it has been suggested that autocrinally secreted IGF-1 stimulates the proliferation of these cells [203]. Another panel of cultured CRC cells (HCT116, HT29, LoVo, WiDr, CoLo201, and LS180) showed that glucose causes an increase in expression of GLUT1, amphiregulin (AREG) (member of epidermal growth factor (EGF) family protein), and HIF-1 luciferase reporter promoter. Inhibition of AREG expression reduced the uptake of glucose and production of lactate [204]. Other studies demonstrated increased expression of e.g., GLUT1, transforming growth factor β1 (TGF-β1), PI3K, Akt, mTOR and Bcl-2 in CRC tissues vs. adjacent normal tissues, with silencing of the GLUT1 gene inhibiting proliferation and promoting apoptosis of CRC cells through inactivation of TGF-β/PI3K/Akt/mTOR signaling [205]. Levels of expression of GLUT4, in greater omental adipose tissue, were lower in MetS and CRC compared to MetS patients without CRC. Reduced GLUT4 expression and elevated ERK and IGF-1 in CRC patients with MetS correlated with CRC clinical characteristics (e.g., size, distant metastases and more advanced tumor stage) [32]. 

With regard to the role of Insulin/IGF system in metabolic reprogramming in CRC, studies on HCT116 cells showed an inducing effect of IGF-1 on the increase in HIF-1α synthesis, the main regulator of the Warburg effect and the well-known VEGF gene transactivator. IGF-1 stimulation of HIF-1α and VEGF mRNA expression was inhibited by cell treatment with PI3K and MAPK signaling pathway inhibitors [206]. Another of the proposed mechanisms driving aerobic glycolysis is the upregulation of a novel gene called colorectal neoplasia differentially expressed (CRNDE), supported by the transcriptomic changes and effect on lactate secretion seen in CRNDE knockdown cells [33]. Elevated levels of the nuclear transcripts of CRNDE promote Warburg effect, by increasing glucose metabolism, lactate secretion and lipid synthesis [33,207]. Insulin/IGF has been shown to repress CRNDE intronic transcripts (gVCIn4 region in cell nucleus) through two signaling pathways, i.e., PI3K/Akt/mTOR and Raf/MAPK. The upregulation of CRNDE in CRC and its downregulation by insulin/IGF seem contradictory but may be connected to different requirements for metabolic processes and cell division. The elevated CRNDE expression potentially required for promoting anabolic pathways in the context of mitogenic activation by Insulin/IGF axis [33]. It could be an independent prognostic factor of poor prognosis for the prediction of the overall survival (OS) of CRC patients. It forms a functional complex with heterogeneous nuclear ribonucleoprotein U-like 2 proteins (hnRNPUL2), directing the transport of this nucleoprotein between the nucleus and the cytoplasm. In the cytoplasm, this protein is an important mediator for inducing CRNDE overexpression by increasing CRNDE stability [207]. CRNDE nuclear transcripts also feedback on upstream Insulin/IGF signaling, but the extent to which these pathways can be weakened probably depends on their mutational status, especially constitutively activating mutations of specific insulin signaling-associated genes [33,207]. 

Recent Wei et al. studies also point to the regulation of glucose metabolism in CRC via IGF signaling with participation of kallikrein-related peptidase (KLK10). Elevated levels of KLK10 proteins were observed in four CRC cell lines (HT29, SW480, DLD1, and HCT116) vs. normal human colorectal epithelial cells (CCD-18Co). Knockdown of KLK10 in HT29 cells drastically reduced their lifespan and caused their apoptosis, as well as inhibited glucose metabolism. Furthermore, it also resulted in silencing of PI3K/Akt/mTOR signaling activation. KLK10 “targeting” inhibited glucose uptake, lactate production and GLUT1 expression. Importantly, the reactivation of the PI3K/Akt/mTOR pathway by IGF-1 significantly reversed the inhibitory effect of KLK10 cessation on CRC cell growth and glucose metabolism. The authors conclude that KLK10 may act as an oncogene to facilitate the development of CRC by increasing cell growth and glycolytic metabolism. This activity is related to the activation of the PI3K/Akt/mTOR pathway [208]. 

**Figure 1:**
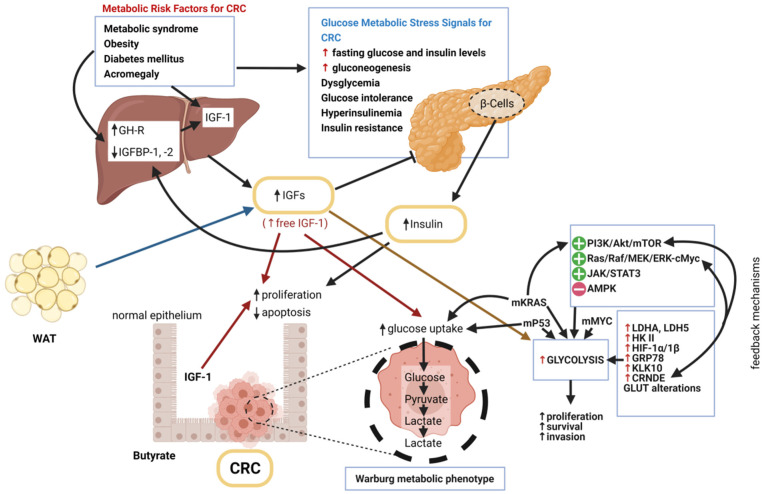
Schematic illustration of the insulin/IGF-1 system involved in the development and progression of colorectal cancer (CRC) through direct (mitotic effects) and indirect (aerobic glycolysis) activity. [↑,↓-increase (up-regulation)/decrease (down-regulation) expression/level; AMPK-adenosine monophosphate (AMP)-activated protein kinase; CRNDE-colorectal neoplasia differentially expressed gene; GLUT-glucose transporter; GH-R-growth hormone receptor; GRP78-glucose-regulated protein 78; HIF-1α, -β-hypoxia-inducible transcription factor-1α, -β; HK II-hexokinase II; IGF-1-insulin-like growth factor 1; IGFBP-1;-2-IGF binding proteins 1,-2; JAK/STAT3-Janus kinase/signal transducer and activator of transcription; KLK10-kallikrein-related peptidase; LDHA, -5-lactate dehydrogenase A, -5; mKRAS-mutant KRAS gene; mMYC-mutant MYC gene; mTP53-mutant tumor protein 53 gene; PI3K/Akt/mTOR-phosphatidylinositol 3 kinase/Akt/mammalian target of rapamycin complex; WAT-white adipose tissue].

The role of insulin/IGF-1 axis in colorectal carcinogenesis, through direct pro-proliferative effects and indirectly through the alterations in glucose metabolism in CRC cells, is presented on Figure 1. 

### 3.3. Genetic Alterations of IGF-1 System Components and Glucose Metabolism in CRC

The role of genetic changes in the IGF-1 gene during the development of CRC is debatable. No links were observed between the CA repeat length, or any of the single nucleotide polymorphisms (SNPs) in the IGF-1 and IGFBP-3 genes, and the risk of German CRC cases [209]. Similarly, there has been no association with the occurrence of polymorphic variations (four SNPs) in IGF-1, IGFBP-3, INSR, the insulin receptor substrate 2 (IRS2) genes and risk of CRC in the Iranian population. The only possible link to the risk of CRC dependent on BMI of the patients would be the presence of the IRS2 variant (rs2289046). However, this requires further research [210]. Serum IGF-1 concentrations were also studied in patients with CRC with distribution of the *IGF-1R* polymorphism +3179G/A (rs 2229765) genotype. Correlation occurred more frequently between the presence of this *IGF-1R* polymorphism, serum IGF-1 concentration and more advanced CRC than cancer in the early stages. Reduced IGF-1 levels have been demonstrated in patients with the GG genotype, with elevated levels for the dominant genotype (AA/AG). Finally, a dominant genetic model was established for the *IGF-1R* polymorphism rs2229765 and CRC progression [211]. 

When it comes to glucose metabolism disorders in CRC, a genetic loci newly associated with increased CRC progression was identified, related to glucose metabolism enzymes and associated with the activity of certain miRNAs, the rs18407893 at 11p15.4 in 3′-UTR LDHA, which maps to the seed recognized sequence by miR-374a. Cancer cells with miR-374a overexpression exhibit reduced LDHA levels compared to miR-374a-MUT (rs18407893 at 11p15.4) [196]. In contrast, the Chinese population showed that for functional *GLUT1* polymorphism (rs710218), people with genotype TT or genotype AT rs710218 had a significantly increased risk of CRC compared to those with homozygous AA. These results suggest that *GLUT1* functional SNP, rs710218 may be CRC risk factor. However, the exact mechanism is not yet known [212]. 

Recent studies de Kort et al. suggest that certain genetic changes in the IGF pathway (IGF-1 19-CA repeat polymorphism) may increase the risk of CRC in subjects with DM II. A combined category comparison, with non-DM II in the lowest GRS tertile as reference, reported that the presence of more unfavorable IGF pathway alleles was connected to increased CRC risk with in both the presence and absence of DM II, with strong increase in CRC risk observed in the presence of DM II [213]. 

## 4. Therapeutic Strategies for Reduction of Metabolic Glucose Disorders in CRC

The high blood glucose levels that occur in obese subjects with MetS, DM II, or prediabetes IR provide a promoting environment for the development and metabolic “engine” of cancer, including CRC [12,13,214]. Numerous studies also indicate a link between hyperinsulinemia, IR and colonic pre- and neoplastic lesions in acromegalic patients [174,176,177,178]. The choice of treatment for acromegaly should take into account the alleviation of glucose metabolism disorders, by reversing IR and reducing gluconeogenesis, e.g., by normalizing GH/IGF-1 levels [175,176]. Recently, it has been proposed to use sodium glucose cotransporter inhibitors (SGLT2is) to treat DM II in acromegaly. Lowering the level of circulating insulin that is unique to this class of therapeutics can have a beneficial role in regulating the GH/IGF-1 axis [215]. 

### 4.1. Therapeutic Agents Regulating Insulin/IGF Signaling

Diabetes mellitus (especially DM II) is one of the diseases that increase the incidence of CRC. Of the antidiabetic drugs, such as insulin, sulfonylureas, dipeptidyl peptidase-4 (DPP4) inhibitors, metformin (MET), and analogs of the insulinotropic GLP-1, the oral antidiabetic drug, MET (1,1-dimethylbiguanide), is the most promising therapeutic for the prevention and treatment of cancer in patients with diabetes, and as a stand-alone anticancer drug [216,217,218,219]. 

As the meta-analysis shows, MET use was associated with a significantly reduced relative risk of CRC in patients with DM II [214,216]. The very beneficial effects of MET as a potential chemotherapeutic and adjuvant agent for CRC with association with DM II have been demonstrated in numerous epidemiological, preclinical, and clinical trials (reviewed in: [219]). 

The direct effect of metformin includes AMPK-dependent and AMPK-independent effects, while glucose levels decrease, hyperinsulinemia and an increase in IGF-1 levels are considered as indirect [218]. AMPK-dependent activity occurs through liver kinase B1 (LKB1), which activates and/or inactivates various signaling targets, e.g., mTOR, PTEN/PI3K/Akt, MAPKs, as well as transcription factors (e.g., NF-κB, FOXO, and p53), calcium/calmodulin-dependent proteinase (CaMKK), and TGF-β-activated protein kinase 1 (TAK1) [219]. By inhibiting IR, MET acts as an inhibitor of epithelial cell growth by reducing the activity of mTOR signaling [217,218]. MET therapy intensifies in vivo apoptosis and impairs the ability of p53-deficit cells to survive in vitro in glucose restriction conditions. Treatment with MET or the second known AMPK signaling activator, 5-aminoimidazole-4-carboxamide ribonucleotide (AICAR), has been shown to reduce tumor growth in p53-deficit HCT116 cells. The authors conclude that MET could be used especially in p53-deficit tumors, which are often resistant to existing forms of chemotherapy (CTX) or radiotherapy (RT) [220]. In turn, mouse model studies demonstrated that MET downregulated proliferation and tumor angiogenesis, as well as augmented the antitumor effect of oxaliplatin [221].

Regarding the IGF-1 system, MET can promote IGF-1R phosphorylation, inhibiting IGF-1 signaling. This results in increased peripheral insulin sensitivity and muscle glucose uptake, while reducing plasma insulin levels and hepatic glucose production. As a result, the activation of IGF-1/IGF-1R is further inhibited, which indirectly leads to an antiproliferative effect in cancer cells [219]. In addition, some studies show that combining MET and insulin to treat DM reduces the detection rate of colon adenomas and is therefore more effective at reducing CRC risk among DM II patients [222]. 

The role of IGF system components in the action of therapeutic agents is also enhanced by current research based on bioinformatical analysis. They concern the action of berberine (BBR), which inhibits proliferation and induces the retention of phase G0/G1 in CRC cells by reducing IGF2BP-3. Disabling IGF2BP-3 may inhibit the PI3K/Akt pathway, resulting in inhibition of cell proliferation and cycle transition [223].

Animal model and in vitro CRC studies also demonstrated MET’s preventive role in diabetes, and indirectly in CRC, through the regulation of expression of enzyme involved in glucose metabolism [224], a topic which will be described later in this work.

### 4.2. Glucose Uptake and Glycolysis Inihbiting Factors 

Since cancer cells are more dependent on glycolysis than normal cells, therapeutic agents that inhibit glycolysis may be more harmful to malignant than non-malignant cells. Therefore, glycolysis suppression is a beneficial therapeutic strategy in the fight against cancer [225,226]. Thus, this mechanism is the form of action of therapeutics studied in recent years, e.g., glucose analogues (e.g., 2-Deoxy-D-glucose) [226,227], plant-based products, e.g., alkaloids (berberine) [223,228,229], rosmarinic acid [230], resweratrol [231], or vitamins (e.g., vitamin C) [232]. The modes of action of functional elements derived from plants (phytometabolites) involved in Warburg effect are already reviewed [233]. Among the numerous effects of MET there are also molecular targets associated with metabolic homeostasis in both diabetic and non-diabetic patients with CRC [219].

In the current work, only the latest therapeutic measures regulating glucose metabolism in CRC were selected for review.

#### 4.2.1. Anti-HIF-1α Factors

Rosmarinic acid (RA) is isolated from herbal balm mint plants, e.g., *Rosmarinus officinalis, Melissa officinalis*, and *Prunella vulgaris L*. In addition to inhibiting HIF-1α, RA reduced glucose consumption and lactate production in CRC cells. Furthermore, it inhibited the activity of pro-inflammatory cytokines and microRNAs associated with inflammation in CRC. Warburg effect inhibition has been shown to occur via miR-155 in the IL-6/signal transducer and activator of transcription 3 (IL-6/STAT3) pathway inactivation mechanism [230].

#### 4.2.2. Anti-Glucose Transporter Factors (Anti-GLUTs)

Yao et al. using oridonin, a natural diterpenoid isolated from *Rabdosia rubescens*, demonstrated deactivation of phospho-AMPK, resulting in down-regulation of AMPK-related GLUT1 and induction of autophagy in the CRC cells. First, anticancer activity of oridonin was demonstrated in vitro and in vivo. Then, using miRNA profiling of SW480 cells, it was shown that oridonin inhibits glucose uptake and reduces lactate exports by significantly reducing GLUT1 and monocarboxylate transporter 1 (MCT-1) levels in vitro and in vivo. Oridonin can therefore affect glucose metabolism, induce autophagy and accelerate cancer cell death through a metabolic pathway [234]. 

Berberine, one of isoquinoline alkaloids from *Coptidis Rhizoma* also inhibits glucose uptake and the transcription of glucose metabolic genes (e.g., GLUT1, LDHA and HK II). The mechanism of its action was based on the inhibition of mTOR-dependent HIF-1α synthesis [228]. 

It is also proposed to combine conventional CTX with metabolic strategies, including vitamin C (vit. C) and other molecules, targeting key Warburg players. The mechanism of action of vit. C in inhibition of the Warburg effect involves induction of RAS detachment from the cell membrane and inhibition of ERK 1/2 phosphorylation and pyruvate kinase muscle isozyme 2 (PKM2)—an isoenzyme of the glycolytic enzyme PK, resulting in a strong decrease in the expression of GLUT1 and PKM2/Polypyrimidine Tract Binding Protein (PTBP) [232]. 

Wu et al. used a galactose-conjugated (trans-R, R-cyclohexane-1,2-diamine)-2-chloromalonato-platinum (II) complex (Gal-Pt) to treat CRC. The therapeutic index was shown to be more than 30-fold higher compared to oxaliplatin. Research also suggests that cellular uptake of Gal-Pt was regulated by GLUTs in HT-29 cells [235]. 

In turn, recent studies of Han et al. also point to the role of a ubiquitin E3 ligase, TRIM29 in colon carcinogenesis by promoting the degradation of pyruvate kinase via the ubiquitin-proteasome pathway. The direct target for TRIM29 is PKM1 to reduce PKM1/PKM2 ratio. These results suggest that TRIM29 as a cancer promoter, particularly in right-sided CRC, may be a potential therapeutic target [236]. 

A combination of actions of a plant-based compound, kaempferol, and miR activity modulation was also demonstrated for the recently described miR-339-5p-hnRNPA1/PTBP1-PKM2 axis, which inhibits glycolysis and CRC growth. Kaempferol, a flavonoid found in a variety of natural foods, exhibits significant inhibitory effects on CRC. It promoted miR-339-5p expression, with its direct targets identified as hnRNPA1 and PTBP1 [237].

#### 4.2.3. Anti-Lactate Dehydrogenase Factors

The potential role of LDHA gene and its isoenzyme, i.e., LDH5 as a prognostic marker in cancer patients, as well as a predictor of response to RT and CTX, or even the main objective in cancer treatment and radiosensitization, is widely discussed (reviewed in: [238]). As previous studies of these authors have shown for CRC, serous concentrations of LDH and LDH5 in tissues are complementary and may play a role in predicting responses to CTX. The addition of vatalinib reduced the effect of tissue LDH expression on the prognosis in patients [198]. In other studies, in patients with metastatic CRC and high LDH levels, the addition of anti-VEGF antibody (bevacizumab) to CTX led to a reduction in disease progression and an increase in PFS [239]. 

Other interesting observations on cultured CRC cells indicate that mild treatment with hyperthermia (HT) accelerates glucose metabolism and induces oxidative stress. This work provided evidence that temperature changes can modulate the metabolism of CRC cells and thus potentially affect treatment outcomes [240]. One of the mechanisms described in HT resistant LoVo cells, is the up-regulation of IGF2BP-1 compared to parental cells. The immediate target of IGF2BP-1 in this study was the LDHA mRNA. The authors conclude that targeting the IGF2BP-1-LDHA-glycolysis pathway may be a promising therapeutic approach to enhance the anticancer effects of HT treatment [241]. 

#### 4.2.4. Anti-Pyruvate-Dehydrogenase (PDH) Complex

In rat model and in vitro studies it has been shown that MET also reduces the disturbed balance in the expression of enzymes involved in glycolysis, e.g., reduces the HK activity, increases PDH activity. The in vitro model showed that the expression of isocitrate dehydrogenase 1 (IDH1)—key enzyme in the TCA cycle increased, PKM2 expression decreased, HK activity gradually decreased and PDH gradually increased with increased MET concentration and treatment time. The ability to inhibit the formation of aberrant crypt foci (ACF) and tumors after MET use was observed. This therapeutic lowered the colon tissue proliferation index and inhibited the growth of cultured cells [224]. 

Other noteworthy therapeutics also include resveratrol (RES), a known plant polyphenol with antioxidant, anti-inflammatory and anti-proliferative properties. It results in induction of both cell growth arrest and a metabolic reprogramming of CRC cells. It reduces glycolysis, in combination with a decreased pentose phosphate activity and increased ATP production. The “metabolic” target of RES is PDH, leading to increased PDH activity. Further studies have shown that RES may improve the oxidative properties of cancer cells by calmodulin kinase kinase B (CamKKB/AMPK) signaling. Resveratrol, on the other hand, did not modulate the levels of LDHA, GLUT1 or the enzyme catalyzing the last stage of glycolysis, PKM2 [231]. 

#### 4.2.5. Anti-Glucose-Regulated Protein 78 (GRP78)

2-Deoxy-D-glucose (2DG), a glucose analogue, acts on glucose metabolism, depriving cancer cells of energy. In addition, 2DG increases oxidative stress, inhibits N-linked glycosylation, and induces autophagy. In cancer therapy, it is usually used in combination with other compounds (reviewed in: [226]). 2DG cell line-specific effects on the survival of different cancer cells (including CRC cells) were also demonstrated. 2-DG induced ER stress, assessed on the basis of accumulation of marker proteins: unfolded protein response (UPR) regulator, ER chaperone and G *Protein-*Coupled Receptor 78/binding immunoglobulin protein (GRP78/BiP) [227]. The GRP78 protein is also regulated by BBR, as shown on CRC cells. Depending on the dose, BBR inhibited the proliferation and migration of cancer cells and induced their apoptosis [229]. 

#### 4.2.6. Other Factors Targeting the Warburg Effect

Other factors of plant origin acting on glucose metabolism in CRC cells include Atractylenolide I (ATL-1). This compound is an eudesmane-type sesquiterpenoid lactone derivative of *Rhizoma Atractylodis macrocephalae,* known in traditional Chinese medicine [242,243]. In addition to inhibiting CRC cell invasion and inducing their apoptosis, ATL-1 also alters glucose metabolism, and suppressed stem-like traits. It acts as an ACT/mTOR signaling inhibitor by lowering the phosphorylation of proteins associated with this pathway. In vivo studies have shown a reduction in tumor weight and volume and confirmed impaired aerobic glycolysis, stemness maintenance and Akt/mTOR activation in colorectal tumors [242]. Other studies have confirmed that ATL-1 anticancer activity in CRC is associated with apoptosis induction and glycolysis suppression in CRC cells through inhibition of Janus kinase 2 (JAK2)/STAT3 signaling. ATL-1 significantly inhibited tumor growth also in in vivo conditions [243]. 

In colon tumors recurrent after resection and adjuvant treatment based on 5-fluorouracil (5-FU), using proteomic analysis (113 proteins related to carbohydrate metabolism and antioxidant pathways), reduced levels of metabolic proteins associated with the tricarboxylic acid (TCA) cycle have been demonstrated. The effectiveness of 5-FU also declined in HCT116 cells. The studies have identified differences in carbohydrate metabolism enzyme expression between poor and good prognosis cancers and as well as determination of a marker of resistance to adjuvant therapy based on 5-FU [244]. 

The in vitro model (DLD-1 and SW-480 cells) recently demonstrated some therapeutic efficacy of a novel glucose-methotrexate (GLU-MTX) conjugate. These results confirmed the hypothesis that GLUT1 is active during the cellular uptake of GLU-MTX, with the absorption of the drug mediated by glucose. This conjugate was about 17-times more preferentially accumulated in SW-480 cells compared to free MTX [245]. 

### 4.3. Selected Warburg Effect Suppressing Non-Coding RNAs

Many microRNAs (miRs) regulating Warburg effect were described in CRC [246,247,248,249,250,251]. Almost a decade ago, it was shown that three miRNAs (miR-124, miR-137 and miR-340), inhibit CRC growth by counteracting the Warburg effect through alternative PKM gene splicing regulation. PKM gene expression is switched from PKM2 to PKM1, which inhibits glycolysis rate but increases the glucose flux into OXPHOS [246]. Regarding miR-124, these results are confirmed in another work demonstrating the anti-cancer effect of this miR via modulating energy metabolism in a cascade of feedback PTB1/PKM1/PKM2 [247]. Subsequent studies of this team have shown that switching PKM gene expression from PKM2 to PKM1 by silencing PTBP1, both in vitro and in vivo, also occurs with participation of miR-1 and miR-133b [248]. In the case of miR-1, it has been shown to also inhibit aerobic glycolysis and cancer cell proliferation by inactivating Smad3. It inhibits interactions between Smad3 and HIF-1α, leading to suppression of Smad3 activation and reduced expression of metabolic enzymes in the Warburg effect, i.e., HIF-1α, HK II and monocarboxylate transporter 4 (MCT4) [252]. In turn, miR-181a, whose expression is increased in CRC tissues, induces metabolic shift in CRC cells by inhibiting PTEN expression, leading to an increase in phosphorylated Akt. The increase in lactate production induced by miR-181a results in increased proliferation of cancer cells. These results indicate the role of miR-181a in CRC cells through the PTEN/Akt pathway [253]. This effect of miR-181a on CRC cells (increased proliferation) was also confirmed [254]. Akt (a serine/threonine protein kinase) is a key Akt signaling protein that is activated upon ligand (e.g., IGF-1) binding to IGF-1R, receptor tyrosine kinase activation and IRS-1 protein phosphorylation. Along with MAPK, and mTOR pathways, it is also one of the major signal transduction pathways that promotes survival and growth in response to extracellular signals [17,111]. In contrast, the PTEN protein is a phosphatase and acts as an inhibitor of the PI3K and Akt kinase pathways. The PI3K/PTEN/Akt signaling pathways apparently also affect glucose uptake via GLUT4 translocation (reviewed in: [255]). 

At the same time, these authors showed that miR-181a inhibition in CRC cells occurs with signal transducer and activator of transcription 1 (STAT1), which regulates the expression of this miR by binding to elements in the miR-181a promoter region. 

Wang et al., on the other hand, demonstrated that numerous miRs (e.g., miR-34a, miR-34c, miR-369-3p, miR-374a, and miR-4524a/b) target LDHA and regulate glycolysis in cancer cells [196]. 

Furthermore, miR-98 has been described to bind HK II. MiR-98 expression in CRC tissues has been shown to decrease compared to adjacent colon tissues. This expression was negatively correlated with HK II expression. HK II has been involved in miR-98-mediated suppression of glucose uptake, lactate production and cell proliferation [249]. In turn, research by Santasusagna et al. shows that miR-328 may also be involved in modulating the Warburg effect in CRC by targeting solute career family 2 member 1 (SLC2A1)/GLUT1. The expression of miR-328 is reduced in patients with CRC, which inversely correlates with the classically described increased expression of SLC2A1/GLUT1 in tumors. miR-328 is potentially capable of inhibiting SLC2A1 and consequently to regulate glycolytic activity of GLUT1 (anSLC2A1-encoded protein) in cancer cells [256].

A link between miR-181b (miR-181b-5p), a protein inhibitor of activated STAT3 (PIAS3) and STAT3 has also been shown. The miR-181b contributes to Warburg effect and colon cancer xenografted tumor growth by targeting PIAS3 [250]. A close link between IGF signaling and STAT3/NANOG/Slug signaling in CRC progression was demonstrated by modulating the properties of CSCs. The transcription factor NANOG has been shown to modulate epithelial-to-mesenchymal transition (EMT) and metastasis of CRC through transcriptional regulation of gene expression of Slug (SNAI2). NANOG was shown to be regulated by the extracellular IGF signaling pathway through STAT3 phosphorylation in CRC [257].

Recent research by Fu et al. points to the role of novel miR-206/hnRNPA1/PKM2 axis in the Warburg effect to modulate CRC progression. Like other miRs, miR-206 overexpression induced the transition from PKM2 to PKM1. A novelty of these studies is the identification of the alternative splicing factor, hnRNPA1, as a direct functional target of miR-206 to reprogram *PKM* alternative splicing. MiR-206 expression directly targets hnRNPA1, inhibiting PKM2 expression to weaken the Warburg effect and CRC cell proliferation [251]. 

A new strategy for the treatment of CRC patients with simultaneous hyperglycemia, may be the use of miR-9 as a tumor-suppressive miR. It has been shown that miR-9 downregulates IGF-1R/Src signaling pathway. In addition, the effects of high glucose on increased proliferation, altered cell morphology, EMT protein expression, and promotion of migration and invasion ability of SW480 (low metastatic potential) and SW620 (high metastatic potential) cells were described. These results provide new evidence that IGF-1R signaling is regulated by hyperglycemia in CRC [258].

The role of several long noncoding RNAs (lncRNAs) was also described in inhibition of Warburg effect in CRCs [33,259,260]. These included maternally expressed gene 3 *(MEG3)* lncRNA, overexpression of which inhibited glycolysis, as well as reduced lactate production in CRC cells. Overexpression of MEG3 induced ubiquitin-dependent degradation of c-Myc and inhibited target c-Myc genes involved in the glycolysis pathway, such as LDHA, PKM2, HK II. MEG3 has also positively correlated with serum vit. D levels in patients with CRC, and could be activated by this vitamin and its receptor (VDR). Treatment with 1.25(OH)2D3 was shown to increase MEG3 expression, while VDR knockdown tolerated the effect of MEG3 on glycolysis. These results indicate that, activated by vit. D, MEG3 inhibits aerobic glycolysis in CRC cells by degrading c-Myc [259]. KCNQ1OT1 is another lncRNA whose high expression promotes colon carcinogenesis by increasing aerobic glycolysis through direct binding and stabilization of HK II. KCNQ1OT1 is also a potential predictive indicator [260]. LncRNAs also include the aforementioned CRNDE transcripts. It is worth noting that this is the first report of a lncRNA regulated by insulin/IGFs axis [33]. 

Selected ncRNAs that play role in glucose metabolism as potential targets for treatment in CRC are presented in Table 3.

### 4.4. Energy Restriction Types and Physical Activity

Warburg effect, related to nutrient oxidation in cancer cells, is also an attractive therapeutic target in CRC. Various caloric restriction-based strategies were undertaken with diets targeting tumor cell metabolism, including CRC cells [261,262]. Calorie restriction and intermittent fasting lower blood insulin levels on an empty stomach [263,264,265]. In contrast, regarding IGFs, intermittent fasting and protein restriction, but not calorie restriction, have been shown to lower their levels [266]. The most recent review by Barrea et al. shows the effects of ketogenic diet (KD) in cancer, including glucose/insulin pathway inhibition mechanisms, as well as oxidative stress, mitochondrial metabolism, or inflammatory process. This diet is characterized by high fat intake, moderate or low protein intake and very low carbohydrate intake (<50 g). The advantages and disadvantages of such a strategy are still discussed [261]. The use of KD in tumors is important for two reasons. It lowers the absorption of carbohydrates, which can lead to cancer cell starvation and apoptosis, while increasing the level of ketone bodies available to produce energy in normal cells, but not in cancer cells characterized by lowered OXPHOS [267].

In CRC prevention, some strategies involving caloric restriction in treatment were tested, with existing examples of clinical trials: NCT00653484 (from 2008) and NCT03595540 (from 2018) **[262]**. For different malignant neoplasm (including CRC) prevention, there are some promising results based on moderate PA, Mediterranean/macrobiotic diet with moderate calorie and protein restriction and metformin (a calorie-restriction mimetic drug) to prevent age-related chronic-diseases (ArcCD) in healthy people with MetS (NCT02960711) (from 2016) [268], and fasting-mimicking diet (FMD) consisting of a 5-day plant-based, low-calorie, low protein, low-carbohydrate diet—NCT03340935 (from 2017) [262]. The FMD is designed to result in fasting-like effects on the serum concentrations of IGF-1, IGFBP-1, glucose, and ketone bodies while maintaining a supply of both macro- and micronutrients to minimize the burden and negative effects of fasting [34]. 

The beneficial effect of PA on reducing CRC risk is also suggested. Among diabetics, compared to people who never or rarely took physical activity, physical activity of more than 7 h/week affected the reduced risk of developing CRC in an age-adjusted and gender-adjusted model. PA was inversely correlated with CRC risk in people without diabetes [269]. The likely mechanism underlying the reverse relationship between PA and CRC survival is a decrease in IGF-1 and an increase in IGFBP-3 [270]. Recent reviews show that the role of PA in preventing the development of CRC is based on a reduction of chronic inflammation, modifications of the intestinal microbiota, and metabolic dysregulation [271,272]. A list of a range of clinical trials (completed, ongoing or discontinued) on CRC and PA was also presented in recent reviews [271]. 

### 4.5. Microbiota and the Warburg Effect

The relationship between diets, gut microbiota, and CRC, along with a list of both dietary factors with CRC risk-enhancing effects, as well as protective activity on colorectal cells are all presented in recent reviews [273,274]. There are also descriptions of the mechanisms of action of probiotics as therapeutic agents in CRC, based on reversing the Warburg effect, as well as modulating the intestinal microbiota and immune response [274]. 

In the context of glucose metabolism disorders, interesting observations came from the research into the mechanisms of action of fiber-rich diets and interaction with gut microbiota in the protection of colonocytes [192,275,276]. These studies were firstly focused on the verification of the so-called BT paradox [192,193]. Butyrate is a short-chain FA (SCFA) produced by bacterial fermentation of dietary fiber in the large intestine. It was hypothesized that BT’s ability to cause specific anti-cancer activity in colon cells was caused by the Warburg effect. Therefore, in conditions where BT is not effectively metabolized in the mitochondria, it accumulates in the nuclei and functions as a histone deacetylase inhibitor (HDAC) to increase the expression of target genes, stimulating histone acetylation, inducing apoptosis and inhibiting cell proliferation. In addition, BT has been shown to increase histone acetylation by metabolizing into acetyl-CoA and stimulating histone acetyltransferase (HAT) activity. Furthermore, the metabolic state of the cell affects intranuclear levels of BT and acetyl-CoA and determines whether BT functions as an HDAC inhibitor or stimulates HAT to epigenetic regulation of the expression of different target genes [192]. Using a gnotobiotic mouse model colonized with wild or mutated strains *of BT-producing Butyrivibrio fibrisolvens* bacteria, fiber has been shown to have a strong suppressive effect on tumors, in a manner dependent on microbiota and BT. In cancerous colonocytes, due to the Warburg effect, BT was less metabolized (diminished oxidation) and accumulated with the consequences described earlier (e.g., stimulation of histone acetylation, increase in apoptosis and inhibition of cell proliferation). This mechanism may also be present in vivo in humans, which has been confirmed by the demonstration of elevated BT levels and histone acetylation in colon adenocarcinomas vs. macroscopically normal colon mucosa. Tumor suppression via BT can also partly occur through promotion of Treg cell differentiation and anti-inflammatory effects in the presence of complex intestinal microbiota [276]. Furthermore, according to the authors, the chemoprevention strategy described above is likely to have fewer side effects than the administration of synthetic HDACi as a chemotherapeutic means [275]. 

Current therapeutic approaches regulating glucose metabolism in CRC are presented in Table 4. 

## 5. Concluding Remarks

Metabolic risk factors such as MetS, obesity, diabetes or acromegaly, both hyperglycemia, chronic hyperinsulinemia and an increase in local expression and/or serous concentrations of IGF-1 all can play an important role in the mechanisms of colon carcinogenesis. The presence of adverse alleles in the IGF pathway may further increase the risk of CRC associated with DM II. 

Tumor growth might be promoted by the direct action of insulin, as an anabolic factor and oncogene, or indirect action through IGF-1. In turn, the action of IGF-1 as a mitotic hormone can be direct (mainly via IGF-1R signaling) or indirect (via GH).

In carcinogenesis (including CRC), the basic metabolic pathways are reprogrammed, resulting in the supply of energy, replenishment of metabolic pathway precursors and equivalent reduction necessary to accelerate tumor growth. In tumor-altered colonocytes, a change in the energy source from butyrate to glucose is observed, resulting in a glycolytic phenotype, which is based on increased aerobic glycolysis (Warburg effect). This effect results in much faster ATP production, necessary in the process of increased cellular proliferation and tumor growth.

The role and mechanisms activating aerobic glycolysis, with the participation of the insulin/IGF-1 axis, are only partially understood. Various in vitro CRC models have shown that IGF-1 can regulate glucose metabolism and affect the Warburg effect. The direct effect of IGF-1 on glucose metabolism in CRC seems to depend on the local action of this hormone. This includes increasing cellular glucose uptake or regulating glucose transport through changes in the expression of certain GLUTs, mainly GLUT1. Studies indicate that gene GLUT1 silencing inhibited proliferation and promoted CRC cell apoptosis by inactivating the TGF-β/PI3K/Akt/mTOR signaling pathway. Similarly, the KLK10 gene knockdown inhibited glucose uptake, lactate production and GLUT1 expression, which was associated with silencing of the PI3K/Akt/mTOR pathway. Another of the proposed mechanisms for driving aerobic glycolysis in CRC is the upregulation of the CRNDE gene, also dependent on the insulin/IGF system. The canonical downstream signaling cascades of insulin and/or IGF, the PI3K/Akt/mTOR and Raf/MAPK pathways inhibited CRNDE nuclear transcripts. However, the latter may cause feedback effects on both signaling routes, depending on the presence of activating mutations in PI3K/MAPK pathway components. Increased CRNDE expression in CRC cells can therefore increase anabolic metabolism (Warburg effect) on its own, but also can reactivate the PI3K/MAPK pathway and insulin/IGF mitogenic functions.

In conclusion, the Warburg effect in CRC is the result of the interaction of many processes, such as: activation of signaling pathways (e.g., PI3K/Akt/mTORC, TGF-β/PI3K/Akt/mTOR, and Raf/MAPK), activation of glucose transporters (mainly GLUT1), and key glycolytic enzymes (e.g., LDHA, LDH5, HK II, and PFKFB3), aberrant expression of oncogenes (e.g., MYC, and KRAS) and overexpression of signaling proteins (e.g., HIF-1α and HIF-1β, TGF-β1, PI3K, ERK, Akt, and mTOR). Most of the above processes are directly or indirectly regulated by IGF-1. 

In treatment strategies that take into account glucose metabolic disorders in CRC, any approach that regulates blood glucose levels is also potentially important in the prevention and treatment of this cancer. Of the factors regulating IGF signaling, the most enthusiasm is focused on a long-used oral antidiabetic drug, metformin, also in combination with insulin, moderate physical activity and mediterranean diet. 

In contrast, current therapeutic strategies that target strictly the dependence of CRC cells on altered energy conversion through the mechanism of aerobic glycolysis rely on targets that inhibit glucose uptake and glycolysis. In addition to metformin, these include glucose analogues, compounds of plant origin, vitamins (e.g., vit. C and vit. D3), but also the use of hyperthermia, selected miRNAs and lncRNAs, energy restriction, physical activity, and fiber-rich diets. There are some descriptions of inhibitory effects on cell proliferation via IGF-1 signaling, exerted by therapeutic agents such as berberine (suppression of PI3K/Akt pathway), atractylenolide I (ATL-1) (suppression of Akt/mTOR pathway), vitamin C (suppression of ERK1/2 phosphorylation) and hyperthermia (concerning IGF2BP1). Of the non-coding RNAs, it may be mentioned miR-9 (inhibition of IGF-1R/Src pathway), miR-181 (a and b) and lncRNA known as CRNDE, which appear to directly or indirectly regulate molecules that are components of IGF-1 signaling in CRC, e.g., PI3K/Akt/mTor and Raf/MAPK (CRNDE transcripts) or closely interact with IGF-1 signaling, e.g., PTEN/Akt pathway, STAT1 (miR-181a) and STAT3 signaling (miR-181b). 

## Figures and Tables

**Table 1:** Summary of the possible association between IGF-1, IGF-1R, and IGFBP-3 and colorectal carcinogenesis in vivo.

IGF Component in Serum (S) and Tissue (T)		Material and Methods	Risk/Incidence of Neoplastic Change/CRC	Serum/Local Tissue Level	Ref.
IGF-1 peptide	S	193 cases; 318 controls; ELISA	↑CRC	↑quintile vs. ↓quintile ^a^ (men)	[112]
		79 cases; 107 early-stage and 90 intermediate/late-stage adenomas; ELISA	↑CRC and large or tubulovillous/villous adenoma; Δearly-stage adenoma	↑tertile vs. ↓tertile (woman)	[115]
		75 cases; 146 controls; immunoradiometric assays	ΔCRC	ΔIGF-1	[118]
		cohort of 93,676 postmenopausal women; 438 incident cases; 816 random subcohort; ELISA	↑CRC	free IGF-1 in multivariate models (woman)	[28]
		764 colon adenomas; 775 controls; ELISA	↑colorectal adenoma	↑IGF-1	[113]
		73 colon and 410 rectal cancers; 650 controls; 120 post-operation colon and 211 rectal cancers; ELISA	CRC vs. control and post-operation cancers	↑insulin, ↑IGF-1, ↑IGF-1/IGFBP-3 ratio	[114]
		95 cases; 48 controls; ELISA	CRC vs. control	↑IGF-1, ↑IGFBP-2, ↑VEGF ^a^	[116]
	T	10 cases; 10 controls; specific immunoassays	CRC vs. control	(+) in small but equal amounts in normal and malignant tissue	[125]
		713 cases; IHC	CRC	(+) in 7.5%; 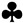 tumor stage (pT1/pT2) and proliferation activity ^a^	[120]
		210 cases; IHC	CRC	(+) in 80% CRC; 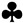 tumor size ^a^; 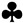 depth of invasion ^a^	[122]
		10 cases; 10 controls; IHC	CRC and control pairs	(−) in all CRCs and controls	[124]
		28 cases; 28 controls; IHC	CRC and control pairs	(+) in 50% CRC; (+) in 39% controls	[121]
IGF-1 mRNA	T	10 cases; 10 controls; Northern blot	CRC vs. control pairs	(+) IGF-1 in CRC and controls; ΔCRC vs. controls	[124]
		10 cases; 10 controls; hybridisation RNase protection assay	CRC and control	(−) IGF-1 mRNA	[125]
		90 cases (63 adenomas and 27 submucosal pT1 cancers); 90 controls; semiquantitative RT-PCR	CRC and control pairs	(+) in 54.4% cases; 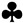 histopathology; (−) in controls or only faintly detected	[123]
		28 cases; 28 controls; real-time PCR	CRC vs. control pairs	↓total IGF-1 mRNA and all mRNA isoforms ^a^	[121]
		202 cases; 202 controls; RT-PCR	CRC vs. control	↓IGF-1 mRNA ^a^	[126]
IGF-1R peptide/mRNA	T	713 cases; IHC	CRC	(+) in 99.6% cases (peptide)	[120]
		90 cases (63 adenomas and 27 submucosal pT1 cancers); 90 controls; RT-PCR	CRC and control pairs	(+) in 37.8% cases; (−) in controls or only faintly detected (mRNA)	[123]
		210 cases; IHC	CRC	(+) in 66% cases (peptide); 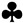 tumor size ^a^; 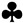 depth of invasion	[122]
		202 cases; 202 controls; RT-PCR	CRC vs. control	↑IGF-1R mRNA ^a^	[126]
IGFBP-3	S	193 cases; 318 controls; ELISA	↑CRC	↓IGFBP-3 ^a^ (men)	[112]
		79 cancers, 107 early-stage and 90 intermediate/late-stage adenomas; ELISA	↑CRC and large or tubulovillous/villous colorectal adenoma; Δearly-stage adenoma	↓IGFBP-3 ^a^ (woman)	[115]
		75 cases; 146 controls; immunoradiometric assays	↑CRC	↑quintile vs. ↓quantile ^a^	[118]
		cohort of 93,676 postmenopausal women; 438 incident cases; 816 random subcohort; ELISA	ΔCRC	ΔIGFBP-3	[28]
		764 colon adenomas; 775 controls; ELISA	Δadenoma	ΔIGFBP-3	[113]
		73 colon and 410 rectal cancers, 650 controls, 120 post-operation colon and 211 rectal cancers; ELISA	CRC vs. control and post-operation cancers	↓IGFBP-3 ^a^	[114]
		95 cases; 48 controls; ELISA	CRC vs. control	↓IGFBP-3 ^a^	[116]
	T	10 cases; 10 controls; IHC	CRC and control pairs	(+) in 7/10 CRC and controls	[124]
		202 cases; 202 controls; RT-PCR	CRC vs. control	ΔmRNA; 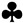 lymph node metastasis; ♦ ↑poor 5-year overall survival	[126]

^a^ statistical significance; ↑—increased/higher; ↓—decreased/lower; Δ-no association/no change; (+)—positive expression; (−)—negative expression; 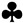
—significant correlation between IGF component and clinical data; ♦—association between IGF component and CRC prognosis; CRC—colorectal cancer; ELISA-enzyme-linked immunosorbent assay; IGF-1-insulin-like growth factor 1; IGFBP-3-IGF binding protein 3; IGF-1R-IGF receptor type I; IHC-immunohistochemistry; RT-PCR-reverse transcription polymerase chain reaction.

**Table 2:** The insulin/IGF-1 signaling and glucose metabolic effects in different research models.

Research Model	IGF-1	Insulin	Glucose Metabolic Effects	Ref.
LID mice	a complete abrogation of liver IGF-1 mRNA; ↓↓(~75%) in cIGF-1	4-fold↑levels; muscle-specific insulin insensitivity	(i) glucose levels were normal vs. control; (ii) abnormal glucose clearance after insulin injection	[8]
LID mice; rhIGF-1 (1 mg/kg); ip for 20 days		↓levels and ↑insulin sensitivity	↓glucose levels to 40% of basal levels	
MID mice	lower mIGF-1; ∼40% ↓of cIGF-1 in *b*MID mice at 4-wk-old mice	↑levels in 8-wk-old male *b*MID, did not change in 12-wk-old *b*MID, and ↓in 16-wk-old *b*MID; ↓in fed *a*MID mice and no response upon food retention; HOMA-IR > 4-fold ↑in male *a*MID mice	(i) m*Igf-1* deletion in male *a*MID mice alters glucose handling and ↑GLUT4 levels; (iii) mIGF-1 modulates anabolism and metabolism in an age-dependent manner; (ii) ↓mIGF-1 progressively disrupts glucose homeostasis in male mice	[80]
WT mice; IGF-1 (1 µg/mL); icv		↑↑levels; ↑insulin sensitivity	(i) ↑food intake; (ii) ↓blood glucose levels; (iii) improves glucose tolerance	[146]
WT mice; anti-IGF-1 Ab; icv		↓levels; normal insulin sensitivity	(i) ↓appetite/food intake; (ii) ↑glucose levels; (iii) normal glucose tolerance	
WT mice; 10^11^ GC/mouse of the AAV-*Igf1* into ARC	↑↑expression in ARC of the hypothalamus	↑↑levels; ↑insulin sensitivity	(i) ↑appetite but unchanged body weight; (ii) ↓blood glucose levels; (iii) improves glucose tolerance	
WT mice *(igf-1*^(+/+)^; untreated Hz mice (Hz, *igf-1*^(+/−)^ and Hz, *igf-1*^(+/−)^ mice treated with IGF-1 (Hz + IGF-1); C	↓cIGF-1 vs. C; ↓*igf*-1 liver expression in untreated Hz groups vs. WT		↑levels of glucose, triglycerides and cholesterol in the untreated Hz group as compared to both C and Hz + IGF-1 groups	[13]
healthy adults; rhIGF-1 (100 µg/kg); iv	↑level 15 min after injection, of which 80% was free IGF-1 (the highest level of free IGF-1 was 350 ng/mL)		(i) the acute hypoglycemia; the lowest blood glucose levels were reached after 30 min: 1.98 ± 0.44 mmol/L; (ii) on a molar basis, was only 6% as potent as insulin in the production of hypoglycemia	[100]
healthy adults; insulin (0.15 IU/kg); iv			(i) the lowest blood glucose levels were reached after 30 min: 1.78 ± 0.29; (ii) inhibits lipolysis more effectively than IGF-1	
healthy adults; rhIGF-1 (20 µg/kg per h); sc; 6 days	↑levels within 2–4 h after starting the infusion, and reached levels of 700 ng/mL after 13–14 h	(i) all fasting values before, during, and after the infusion remained within normal limits; (ii) ↓insulin secretion	Blood glucose remained within normal limits (between 3.7 and 4.4 mmol/L) throughout the study	[169]
healthy adults; high (30 µg/kg) and low (5 µg/kg) doses of rhIGF-1 iv per h; high (23 nmol/kg), and low (0.04 nmol/kg) doses of insulin iv per h	↑total IGF-1 during infusion to 360% of baseline level at the end of high doses and to 150% after low doses	↑levels during high and low insulin doses 5.6- and 1.6-fold above baseline values, whereas they ↓by 25 ± 5 and 22 ± 4% during high and low IGF-1 doses, respectively	(i) glucose rate of disappearance ↑from baseline by 239 ± 16% with high IGF-1 dose vs. 197 ± 18% with insulin iv; (ii) hepatic glucose ↓production by 37 ± 6% during high dose IGF-1 vs. 89 ± 13% during insulin iv	[103]
healthy adults; rhIGF-1 (7 and 14 µg/kg); iv per h during standard OGTT and MTT, respectively	↑total and free cIGF-1 within 10 h after I infusion; on day 2, cIGF-1 were 3.9 and 4.4 times (total), and 1.8 and 4.1 times (free), respectively, above starting levels	(i) ↓insulin by direct suppression of its secretion; (ii) ↓insulin/glucose-ratio; (iii) ↑insulin sensitivity	(i) glucose tolerance remained unchanged in the face of ↓insulin	[104]
nondiabetic subjects with a wide range of BMI		(i) cIGF-1 negatively correlates with IVGTT-derived and OGTT-derived indexes I- and II phase insulin secretion; (ii) ↓cIGF-1 is associated with ↓insulin sensitivity	(i) cIGF-1 positively correlates with glucose disposal; (ii)low cIGF-1 is associated with obesity-related changes, MetS, glucose intolerance, and the development of DM II	[15]
rats; STZ-induced DM	↓cIGF-1; ↓↓IGF-1a/b mRNAs in liver, kidney, and lung tissues; treatment with insulin for 1 wk restored both IGF-1 mRNAs content toward that present in tissues of nondiabetic rats			[167]
DM I subjects (age 13–24 yrs) rhIGF-1/IGFBP-3 complex; 2 days; sc; two groups and placebo	cIGF-1 levels were in the physiological range	↑insulin sensitivity following the two highest doses of rhIGF-1/IGFBP-3, whereas the lower doses had little effect on insulin sensitivity	Enhances glucose metabolism by controlling both endogenous glucose output and peripheral glucose uptake	[105]
(i) nondiabetic subjects; (ii) subjects with impaired glucose tolerance; (iii) DM II subjects	↓cIGF-1 in subjects with MetS vs. subjects without MetS	(i) cIGF-1 positively correlates with HOMA-S; (ii) cIGF-1 independently correlates with insulin sensitivity	cIGF-1 levels are independently related with other components of MetS (impaired glucose regulation)	[12]
DM I subjects; rhIGF-1 (40 µg/kg); sc; basal insulin infusion iv and a hyperinsulinemic clamp	↑cIGF-1 with max 4 h after the injection (398.2 ± 34.9 ng/L)	↑level during the hyperinsulinemic euglycemic clamp	(i) ↓hepatic glucose production rate; (ii) ↑peripheral glucose uptake; (iii) direct effect on glucose and protein metabolism and acts together with insulin	[64]
obese subjects with DM II and with IR; rhIGF-1 (100 µg/kg); sc for 6 wks	↑cIGF-1 was accompanied by a ↑IGFBP-2, slight ↓IGFBP-3, and ↑IGFBP-1	↓mean levels from 108.0 to 57.0 pmol/L during the modal day measurements and from 97.2 to 72.0 pmol/L during the MMT	(i) ↓blood glucose; (ii) ↑insulin sensitivity; (iii) improves glycemic control in DM II were associated with ↓insulin levels	[106]

↑, ↓—increase (up-regulation)/decrease (down-regulation, low) expression/level; ↑↑, ↓↓—marked increase/decrease; AAV—adeno-associated virus; Ab—antibody; ARC—arcuate nucleus of the hypothalamus; C—control; DM (I, II)—diabetes mellitus (type I, II); GLUT4—glucose transporter 4; HOMA-(IR, S)—homeostatic model assessment-insulin resistance, -sensitivity; h—hour(s); Hz—heterozygous; icv—intracerebroventricular injection; (c, m)IGF-1-(circulating, muscle) insulin-like growth factor 1; IGFBP-1;-2;-3—IGF binding proteins 1,-2,-3; ip—intraperitoneally; IR—insulin resistance; iv—intravenous; IVGTT—intravenous glucose tolerance test; LID—liver IGF-1—deficient mouse model; (*a, b*)MID-(adult, birth) muscle-specific IGF-1-deficient mice; MetS—metabolic syndrome; MTT—meal tolerance tests; OGTT—oral glucose tolerance test; rh—recombinant human; sc—subcutaneous; STZ—streptozocin; wk(s)—week(s); WT—wild-type; yrs—years.

**Table 3:** Examples of non-coding RNAs that play role in glucose metabolism as potential targets for treatment in colorectal cancer.

Non-Coding RNA		Research Model	Mechanism of Change in Function	Ref.
miRNAs	miR-124, miR-137, miR-340	HCT116, DLD1, SW480 and HT29 cells; CRC tissues	(i) switch PKM gene expression from PKM2 to PKM1; (ii) ↓glycolysis rate, but ↑the glucose flux into oxidative phosphorylation	[246]
	miR-124	CRC cells; xenografted mice; CRC tissues	(i) ↓in CRC and adenoma tissues vs. adjacent tissue (ii) acts as a tumor-suppressor; (iii) ↑apoptosis and/or autophagic survival; (iv) targets *PTB1* through the switching of PKM isoform expression from PKM2 to PKM1	[247]
	miR-181a	HCT15 and HCT116 cells; CRC tissues	(i) ↑in CRC tissue; (ii) ↑cell proliferation through ↑glycolysis; (iii) suppressed PTEN expression by targeting its 3′-UTR, thus resulting in ↑Akt phosphorylation; (iv) causes an ↑lactate production and ↑cell proliferation through the PTEN/Akt pathway	[253]
		LoVo and SW480 cells	(i) ↑cell proliferation through PTEN; (ii) ↑PTEN in response to STAT1 overexpression or miR-181a inhibition; (iii) ↓PTEN in response to STAT1 knockdown or miR-181a overexpression	[254]
	miR-1, miR-133b	DLD-1 cells and WiDr cells; xenografted mice; CRC tissues	(i) ↓in CRC and adenomas vs. C tissue; ↑in C tissue except muscle; (ii) induces growth suppression and autophagic cell death through the switching from PKM2 to PKM1 by silencing PTBP1 expression; (iii) ↑↑PTBP1 expression in CRC and adenomas	[249]
	miR-1	HCT116, SW480, SW620, HT-29 cells; mice	(i) suppresses aerobic glycolysis and tumor cell proliferation via inactivation of Smad3 and targeting HIF-1α, leading to ↓HK II and ↓MCT4 expression (ii) Smad3 was central to the effects of miR-1 in CRC	[252]
	miR-98	SW480 and HCT116 cells; CRC tissues	(i) ↓in CRC vs. C tissue; (ii) inhibits glycolysis by targeting HK II; (iii) negatively correlates with HK II expression in CRC tissues	[249]
	miR-181b (miR-181b-5p)	HCT116, HT-29, HEK-293T cells; xenografted mice	(i) is a direct regulator of PIAS3; (ii) promotes the Warburg effect and the growth of colon cancer xenografts; (iii) interacts with STAT3 phosphorylation in a positive feedback loop in CRC cells via regulating PIAS3 expression	[250]
	miR-206	CRC cells	(i) ↓in CRC vs. C tissue; (ii) negative correlation with S, and inverse correlation with OS; (iii) causes ↓the cell proliferation, glucose consumption and lactate production; (iii) overexpression induces switching from PKM2 to PKM1; (iv) hnRNPA1 is a direct target of this miR to suppress PKM2 expression	[251]
	miR-34a, miR-34c, miR-369-3p, miR-374a, miR-4524a/b	HCT116, HCT15, HT29, Panc-1, Bxpc-3, CFPAC-1 cells; TMA with CRC; tumor bearing mice	(i) target LDHA and regulate glycolysis (ii) negatively correlates with LDHA expression in CRC tissues; (iii) a genetic loci newly associated with ↑CRC progression, rs18407893 at 11p15.4, which maps to the seed sequence recognized by miR-374a	[196]
	miR-328	LOVO and SW480 cells; CRC tissues	(i) ↓in CRC vs. C tisuse; (ii) directly targets SLC2A1 3′-UTR; (iii) inhibits SLC2A1 and regulates GLUT1-mediated glycolytic activity in cancer cells	[256]
	miR-9	SW480 and SW620 cells; CRC tissues	(i) ↓in CRC with hyperglycemia and with high levels of CEA; (ii) causes ↓IGF-1R/Src signaling and downstream cyclin B1 and N-cadherin, but ↑E-cadherin; (iii) high glucose level promoted cell proliferation, migration, and invasion ability of the cells, ↑G1 population, and EMT protein expression	[258]
lncRNAs	CRNDE transcripts	HCT116, HT29, LS174T cells	(i) regulate genes involved in glucose and lipid metabolism; (ii) promote the metabolic changes by which cancer cells switch to aerobic glycolysis; (iii) are regulated by insulin/IGFs; (iv) downstream PI3K/Akt/mTOR and Raf/MAPK pathways repress CRNDE nuclear transcripts	[33]
	MEG3	DLD-1 and RKO cells; CRC tissues	(i) overexpression causes ↓glycolysis, and ↓lactate production in CRC cells; (ii) ↑degradates of c-Myc and ↓c-Myc target genes such as LDHA, PKM2 and HK II; (iii) can be activated by vit. D and VDR; (iv) vit. D-activated *MEG3* causes ↓aerobic glycolysis in CRC cells *via* degradation of c-Myc	[259]
	KCNQ1OT1	SW48, LoVo, HCT116, SW620, HT-29, RKO cells; CRC tissues	(i) ↑in CRC vs. C tissues; (ii) ↑expression correlates with poorer prognosis in patients; (iii) ↑CRC cell proliferation by ↑aerobic glycolysis; (iv) directly binds to HK II; (v)correlates positively with HK II expression and prognosis in CRC	[260]

↑,↓—increase (up-regulation)/decrease (down-regulation, suppression); C—control; CRC—colorectal cancer; CRNDE—colorectal neoplasia differentially expressed gene; GLUT—glucose transporter; EMT—epithelial-to-mesenchymal transition; HIF-1α—hypoxia-inducible transcription factor-1α; HK II—hexokinase II; hnRNPA1—heterogeneous ribonucleoprotein A1; IGFs—insulin-like growth factors; LDHA—lactate dehydrogenase A; lncRNA—long-non coding RNA; MCT4—monocarboxylate transporter 4; MEG3—maternally expressed gene 3; miR—microRNA; MTX—methotrexate; OS—overall survival; PDH—pyruvate dehydrogenase complex; PFS—progression-free survival; PIAS3—protein inhibitor of activated STAT3; PI3K/Akt/mTOR—phosphatidylinositol 3 kinase/Akt/mammalian target of rapamycin complex pathway; PKM 1,2—pyruvate kinase in muscle 1, 2; PTB1—polypyrimidine tract binding protein 1; PTEN—phosphatase and tensin homolog on chromosome ten; Raf/MAPK—raf kinase/mitogen-activated kinase pathway; S—tumor stage; SLC2A1—solute career family 2 member 1; STAT3/JAK2—signal transducer and activator of transcription3/Janus kinase2; TMA—tissue microarray; UTR—untranslated region; vit.—vitamin; VDR—vit. D receptor.

**Table 4:** Examples of therapeutical options for anti-aerobic glycolysis (Warburg effect) in colorectal cancer.

Targeted Agent	Model of the Study	Results	Role in CRC	Ref.
PTK787/ZK 222,584 (vatalanib)	CRC tissues; LDH serum; IHC method	(i) ↑LDH5 related to poor PFS only in the placebo group; (ii) vatalanib improved response and PFS in this group	predicting the response to CTX	[198]
CTX + bevacizumab and CTX only; NCT01878422	mCRC patients	in patients with ↑LDH, the addition of bevacizumab to CTX led to a significant ↓in the rate of progressive disease and to a prolonged PFS	phase III prospective multicentre randomized ITACa	[239]
GLU-MTX	DLD-1 and SW-480 cells	(i) ↓cell viability (DLD-1); (ii) 17-fold ↑uptake of GLU-MTX in tumor cells vs. MTX (SW-480); (iii) cleavable linkage allows intracellular MTX release after selective uptake through GLUT1	may offer a better tumor selectivity, growth inhibition at reduced toxicity	[245]
2-DG	HTC116 and RKO cells	(i) triggers ER stress; (ii) in HCT116 cells ER stress stimulates autophagy	preventive/prospective	[227]
Gal-Pt; oxaliplatin	xenograft tumor model; HT-29 cells	(i) ↑therapeutic index by over 30-fold compared to cisplatin and oxaliplatin; (ii) the uptake of Gal-Pt was regulated by glucose transporters	preventive/prospective	[237]
TRIM29	SW480 and HT29 cells; CRC tissues	(i) ↑in CRC vs. control; (ii) associated with poor clinical outcome; (iii) promotes the malignant phenotype in vitro and in vivo; (iv) promotes mainly PKM1 degradation; (v)directly targets PKM1 to ↓PKM1/PKM2 ratio	preventive/prospective	[236]
Resweratrol	Caco2 and HTC116 cells	(i) ↑PDH activity; (ii) ↑oxidative capacities and ↓glycolysis, in association with a ↓pentose phosphate activity and an ↑ATP production	preventive/prospective	[231]
Berberine	SW480 and HT-29 cells	(i) ↓cell proliferation and migration; (ii) ↑cell apoptosis, in a dose-dependent manner; (iii) ↓expression of GRP78	preventive/prospective	[229]
	HCT116 and KM12C cells	↓glucose uptake and the transcription of GLUT1, LDHA and HK II genes	preventive/prospective	[228]
	bioinformatical analysis	(i) ↓cell proliferation and ↑G0/G1 phase arrest in CRC cells by ↓IGF2BP-3; (ii) knockdown of IGF2BP-3 could suppress the PI3K/Akt pathway to ↓cell proliferation	preventive/prospective	[88]
Rosmarinic acid	CRC cells	(i) ↓glucose consumption and lactate generation; (ii) inhibits expression of HIF-1α; (iii) ↓the cytokines and miRNAs related to inflammation	preventive/prospective	[230]
Kaempferol	HCT116 and DLD1 cells	(i) ↓proliferation of cells, delayed G1 phase progression and ↑apoptosis; (ii) impaires glucose consumption, which causes ↓lactic acid accumulation and ATP production; (iii) promotes the expression of miR-339-5p with hnRNPA1 and PTBP1 as two direct targets	preventive/prospective	[237]
Atractylenolide I	COLO205 and HCT116 cells; mouse xenograft model	(i) inhibits invasion of cells by ↑apoptosis; (ii) alters glucose metabolism; (iii) suppresses stem-like traits; (iv) ↓Akt/mTOR; (v)↓tumor weight and volume	preventive/prospective	[242]
	HCT116 and SW480 cells; male BALB/c nude mice injected with HCT116 cells	(i) ↓cell viability and colony formation; (ii) ↑cell apoptosis (iii) ↓cell glycolysis; (iv) inhibits STAT3/JAK2 activation	preventive/prospective	[243]
Vitamin C	*KRAS* Mut CRC tissues; SW480, LoVo (*KRAS* Mut, G12V and G13D cells) and HCEC (*KRAS* WT) cells	(i) inhibits ERK 1/2 and PKM2 phosphorylation; (ii) ↓GLUT-1 and PKM2-PTBP dependent protein expression	preventive/prospective	[232]
Metformin (MET)	DMH-induced CRC in diabetic SD rats; LoVo and HT-29 cells	(i) inhibits the formation of ACF/tumors; (ii) inhibits cell growth and ↓the imbalance in the expression of the enzymes involved in glycolysis and the TCA cycle	preventive/prospective	[224]
	HCT116 p53^+/+^ and p53^−/−^ cells; both HCT116 cells inoculated of nude mice	(i) ↓the tumor growth of xenografts; (ii) ↓mitochondrial electron transport; (iii) ↑p53-dependent autophagy; (iv) ↑a metabolic conversion that p53^−/−^ cells are unable to do	preventive/prospective	[220]
MET; oxaliplatin; MET + oxaplatin	DMH-induced CRC in diabetic and non-diabetic mice	(i) ↑in angiogenic and cell proliferation markers; (ii) greater immunostaining for IGF-1R and CD34 in the colon of diabetic vs. non-diabetic mice	preventive/prospective	[221]
NCT02960711 (MET + moderate physical activity + Mediterranean-macrobiotic diet)	both sex with MetS; MET (1700 mg/day) + moderate PA, placebo + moderate PA, MET alone, and placebo	The Me.Me.Me. trial is ongoing. No patient has completed the 5 years of follow-up	preventive/prospective; phase III randomized controlled trial	[268]
Low- or high-fiber diets	BALB/c inbred mice associated with 4 commensal +/− the butyrate-producing *B. fibrisolvens*	A high-fiber diet protects against CRC tumors in a microbiota- and butyrate-dependent manner	preventive/prospective	[276]
Hyperthermia (HT)	SW480, HCT116, and Pt. 93 cells at 32 °C, 37 °C and 42 °C	Provides valuable insights for the metabolic and bioenergetic changes of CRC cells under hypothermia and HT conditions	preventive/prospective	[240]
	HT resistant (HTR) LoVo cells	(i) ↑IGF2BP-1 in HTR cells vs. parental cells; (ii) LDHA mRNA was identified as an IGF2BP-1 direct target; (iii) inhibiting the IGF2BP-1-promoted glycolysis causes sensitisation of CRC cells to HT treatment	preventive/prospective	[241]

↑,↓—increase (up-regulation)/decrease expression/level; ACF—aberrant crypt foci; CTX—chemotherapy; (m)CRC-(metastatic) colorectal cancer; 2-DG-2—deoxy-D-glucose; DMH—dimethylhydrazine; EDG-4,6-O—ethylidene-α-D-glucose, a GLUT1 inhibitor; ER—endoplasmic reticulum; FMD—fasting mimicking diet; Gal-Pt—galactose-conjugated (trans-R,R-cyclohexane-1,2-diamine)-2-chloromalonato-platinum(II) complex; GLU-MTX—glucose-methotrexate conjugate; GLUT1—glucose transporter 1; GRP78—glucose-regulated protein 78; HIF-1α—hypoxia-inducible transcription factor-1α; HK II—hexokinase II; hnRNPA1—heterogeneous ribonucleoprotein A1; IHC—immunohistochemistry; IGF-1R—insulin-like growth factor receptor type I; IGF2BP-1, -3-IGF2 binding protein 1, -3; ITACa—italian trial in advanced CRC; KD—ketogenic diet; LDH5—lactate dehydrogenase 5; MetS—metabolic syndrome; MiR—microRNA; MTX- methotrexate; Mut—mutant; OS—overall survival; PDH—pyruvate dehydrogenase complex; PFS—progression-free survival; PIAS3—protein inhibitor of activated STAT3; PI3K/Akt—phosphatidylinositol 3 kinase/Akt pathway; PKM 1,2—pyruvate kinase in muscle 1, 2; PTBP1—polypyrimidine tract binding protein 1; SLC2A1—solute career family 2 member 1; STAT3—signal transducer and activator of transcription3; TCA—tricarboxylic acid; TRIM29—tripartite motif-containing protein 29; WT—wild-type.

## Data Availability

Not applicable.

## Abbreviations

AICAR	5-aminoimidazole-4-carboxamide ribonucleotide
Akt	serine/threonine-protein kinase or protein kinase B (PKB)
AMPK	adenosine monophosphate (AMP)-activated protein kinase
APC	adenomatous polyposis coli
BMI	body mass index
BT	butyrate
CaMKK	calcium/calmodulin-dependent proteinase
CamKKB	calmodulin kinase kinase B
CI	confidence interval
cIGF-1	circulating IGF-1
CIN	chromosome instability
CoSCs	colonic stem cells
CRC	colorectal cancer
CRNDE	colorectal neoplasia differentially expressed gene
CSM	cancer-specific mortality
DM I, II	diabetes mellitus type 1, type 2
DPP4	dipeptidyl peptidase 4
ENO1	enolase 1
FOXO	forkhead box protein
ERK	extracellular signal-regulated kinase
GA	glutaminase
GH	growth hormone
GLP-1	glucagon-like peptide-1
GLUTs	glucose transporters
GPI	glycosylphosphatidylinositol
GRP78/BiP	G *protein*-coupled receptor 78/binding immunoglobulin protein
HIF-1α	hypoxia-inducible transcription factor 1 alpha
HK II	hexokinase II
HOMA-IR	homeostatic model assessment-insulin resistance
HR	hazard ratio
IGF-1, -2	insulin-like growth factor 1, -2
IGFBPs	IGF binding proteins
IGF-1R	IGF receptor type 1
INSR	insulin receptor
IR	insulin resistance
JAK	Janus kinase
KLK10	kallikrein-related peptidase 10
KRAS	oncogene found in Kirsten rat sarcoma virus
LDH5, A, B	lactate dehydrogenase 5, A, B, etc.
LKB1	liver kinase B1 or serine/threonine kinase 11 *(**STK11**)*
MetS	metabolic syndrome
MYC	family of regulator genes/protooncogenes that code for transcription factors
NF-κB	nuclear factor-kappa B
OD	odds ratio
OXPHOS	oxidative phosphorylation
PDH	pyruvate dehydrogenase complex
PEPCK	phosphoenolpyruvate carboxykinase
PFKM	ATP-dependent 6-phosphofructokinase, muscle type
PKM2	pyruvate kinase muscle isozyme 2
PTBP1	polypyrimidine tract binding protein 1
PTEN	phosphatase and tensin homolog deleted on chromosome ten
ROS	reactive oxygen species
RR	relative risk
SNPs	single nucleotide polymorphisms
STAT1/3	signal transducer and activator of transcription 1/3
STZ	streptozocin
TAK1	TGF-β-activated protein kinase 1
TGF-β	transforming-growth factor beta
TMEM219	the IGFBP-3 receptor
TP53	tumor protein 53
UPC1	uncoupling protein 1; thermogenin
VEGF	vascular endothelial growth factor
